# Reduced insulin use and diabetes complications upon introduction of SGLT-2 inhibitors and GLP1-receptor agonists in low- and middle-income countries: A microsimulation

**DOI:** 10.1371/journal.pmed.1004559

**Published:** 2025-04-17

**Authors:** Global Health & Population Project on Access to Care for Cardiometabolic Diseases (HPACC)

**Affiliations:** University of Malaya: Universiti Malaya, MALAYSIA

## Abstract

**Background:**

Diabetes mellitus, particularly type 2 diabetes, is a growing health concern in low- and middle-income countries (LMICs). The potential impact of newer diabetes medications, such as glucagon-like peptide 1 (GLP-1) receptor agonists and sodium-glucose co-transporter-2 (SGLT-2) inhibitors, on insulin dosage and health outcomes in these settings is not well understood.

**Methods and findings:**

We developed a microsimulation model to estimate the impact of treating patients with type 2 diabetes who use insulin with GLP-1 receptor agonists or SGLT-2 inhibitors in LMICs. The model utilized data from the Global Health and Population Project on Access to Care for Cardiometabolic Diseases (HPACC) dataset, encompassing surveys from 79 countries and clinical trial data to estimate insulin dose reduction. We incorporated weight-based insulin dosing formulas and hazard ratios for severe hypoglycemia, cardiovascular and renal outcomes, side effects of new therapies, and mortality. The primary outcome was the change in insulin dosage, and secondary outcomes were disability-adjusted life years (DALYs) lost per 1,000 person-years by diabetes complication (micro- and macro-vascular).

Our results indicate that the addition of GLP-1 receptor agonists or SGLT-2 inhibitors could reduce insulin dosage by 8.2 IU/day (IQR: 6.9, 9.5) and 5.3 IU/day (IQR: 4.5, 6.2), respectively. The median DALYs lost per 1,000 person-years decreased from 2.20 (IQR: 1.49, 4.02) to 1.01 (IQR: 0.61, 1.86) with GLP-1 receptor agonists and 1.25 (IQR: 0.81, 2.29) with SGLT-2 inhibitors. Primary benefits arose from weight loss, decreased cardiorenal disease, and decreased mortality, with smaller DALY benefits from the prevention of severe hypoglycemia. Key limitations include the inability to differentiate between type 1 and type 2 diabetes in some datasets and reliance on assumptions from clinical trials conducted primarily in high-income countries.

**Conclusions:**

The introduction of GLP-1 receptor agonists and SGLT-2 inhibitors for managing type 2 diabetes in LMICs could significantly reduce insulin dosage and associated health risks, leading to improved outcomes and reduced disability. These findings suggest that expanding access to these newer diabetes medications in LMICs could have substantial public health benefits.

## Author summary

Why was this study done?

What did the researchers do and find?

What do these findings mean?

## Abstract 3

In this microsimulation study, Sanjay Basu and colleagues assess the potential impact of newer diabetes medications, such asGLP-1 receptor agonists and SGLT2 inhibitors, on insulin dosage and health outcomes in low- and middle-income countries.

- People with diabetes in low- and middle-income countries (LMICs) often face challenges managing their condition with insulin, including difficulties with storage, monitoring blood sugar, and the risk of dangerously low blood sugar levels.
- Newer diabetes medications that have a lower risk of causing low blood sugar and further benefit cardiovascular and renal outcomes are becoming available in these countries, but their potential implications for insulin use in LMICs are not well understood.
- This study aimed to estimate how these newer medications could help reduce insulin needs and improve health outcomes for people with diabetes in LMICs.
- We used mathematical modeling with data from 79 countries to estimate how newer diabetes medications (GLP-1 receptor agonists and SGLT-2 inhibitors) could affect insulin use and health outcomes.
- We found that these medications could reduce daily insulin needs by 5–8 units per person while also reducing complications from diabetes.
- The greatest benefits came from weight loss, reduced heart and kidney disease, and lower death rates, with additional benefits from preventing severe low blood sugar episodes.
- These results suggest that making newer diabetes medications available in LMICs could substantially improve the lives of people with diabetes by reducing their reliance on insulin and lowering their risk of complications.
- Healthcare systems in these countries may want to consider increasing access to these medications as they become more affordable and available in generic forms.
- The main limitations of our study include difficulty distinguishing between different types of diabetes in some datasets and relying on assumptions from clinical trials that were mainly conducted in wealthy countries.

## Introduction

Cardiometabolic diseases, including type 2 diabetes mellitus, represent an increasing global health concern, particularly in low- and middle-income countries (LMICs) [1]. The burden of type 2 diabetes mellitus is exacerbated by limited healthcare resources, inadequate access to medications and diagnostics, and insufficient healthcare infrastructure [2]. As the prevalence of type 2 diabetes mellitus continues to rise (with >95% of people with diabetes in LMICs being considered type 2 [3]), there is an urgent need for effective and accessible treatment options that can mitigate the associated complications, minimize the treatment burden, and improve patient outcomes.

One of the critical challenges in managing diabetes in LMICs is the use of insulin therapy, particularly given the complexity of care for monitoring glucose levels, insulin dose adjustments and shortages, poor access to refrigeration and glucose self-monitoring devices and test strips, and high rates of severe hypoglycemia in the context of food insecurity in many LMICs [4]. There has been a shift from the traditional glucose-centric to a cardiorenal-metabolic approach to type 2 management in high-income countries over the past few years, and similarly, there is the question of whether a parallel approach should be considered in LMICs. Cardiovascular and renal comorbidities of diabetes are increasing in LMICs [5,6] and newer medications—specifically, sodium-glucose co-transporter-2 (SGLT-2) inhibitors and glucagon-like peptide-1 (GLP-1) receptor agonists—help mitigate these comorbidities more effectively than insulin alone [7]. A comprehensive network meta-analysis of 764 trials including 421,346 participants has confirmed the superior cardiometabolic benefits of these newer agents compared to traditional therapies [8], with particularly strong effects among those with established cardiovascular disease [9]. Research has also shown that SGLT-2 inhibitors and GLP-1 receptor agonists reduce the dosages of insulin needed among people with type 2 diabetes [10].

The prices of SGLT-2 inhibitors and GLP-1 receptor agonists could be lowered, potentially making them more accessible in LMICs [11]. Recent initiatives by international organizations and pharmaceutical companies aim to reduce the cost of these medications through price negotiations and the introduction of generic versions [12,13]. However, despite these efforts, many ministries of health and policymakers in LMICs remain uncertain about the value of increasing the availability and use of these therapies. Concerns about cost-effectiveness, long-term benefits, and the feasibility of integrating these treatments into existing healthcare systems contribute to this uncertainty [14]. In previous work, we estimated the cost thresholds needed for such therapies to be cost-effective [15]. We also recognize that the pricing, refrigeration requirements, and availability of GLP-1 receptor agonists may be improved by newer GLP-1 receptor agonists that are oral small molecules [16].

Recent policy efforts have monitored the growing adoption of SGLT-2 inhibitors and GLP-1 receptor agonists in LMICs. However, several analysts question whether this adoption will effectively address challenges related to insulin use, such as insulin shortages, refrigeration/equipment limitations, and hypoglycemia, among other challenges in these countries. The global DISCOVER prospective observational study, encompassing 37 countries, reported a substantial increase in the use of SGLT2-is and GLP-1RAs between 2014 and 2016, with median usage reaching 19.4% of people with diabetes by 2016 [14]. In China, a large-scale study revealed a 140-fold increase in SGLT2-i utilization and a 6.5-fold increase in GLP-1RA use between 2018 and 2021 [17]. Médecins Sans Frontières has indicated plans to incorporate the medications into their treatment algorithms for humanitarian settings [18], and several countries are adding them to national formularies, particularly as generics (e.g., for dapagliflozin and liraglutide) and compounded versions become more widely available and lower in cost in LMICs [17].

As access to these medicines continues to expand, questions remain around how beneficial they are for people with type 2 diabetes in LMICs. This study aimed to estimate the potential impact of SGLT-2 inhibitors and GLP-1 receptor agonists on insulin use and DALYs in LMICs. Specifically, we sought to understand how introducing these therapies could reduce insulin dosage and its associated risks and morbidities thereby improving overall health outcomes and reducing disability.

## Methods

### Study design

This study aimed to estimate the potential impact of treating patients in LMICs who have type 2 diabetes and are using insulin with SGLT-2 inhibitors or GLP-1 receptor agonists. We project the change in daily dosage of insulin, associated side effect risks (including reduced severe hypoglycemia, but increased risk of gastrointestinal or urogenital side-effects on the newer medicines), and disability-adjusted life years (DALYs) across countries on data obtained from the Global Health and Population Project on Access to Care for Cardiometabolic Diseases (HPACC) survey dataset [19]. The study focused on LMICs and employed a microsimulation model.

#### Microsimulation model design.

We chose a microsimulation approach over traditional regression analysis for several key reasons. First, microsimulation allows us to model longitudinal aspects of competing risks between diabetes complications, non-diabetes morbidity, and mortality. Second, it enables simulation of counterfactual scenarios that cannot be directly observed in real-world data. Third, it preserves individual-level correlations between variables while incorporating multiple data sources.

The model consists of four interconnected modules:

1. *Population module:* Generates synthetic populations based on HPACC survey participants, preserving joint distributions of age, sex, weight, clinical parameters, and country-specific characteristics.
2. *Insulin dosage module:* Estimates baseline insulin requirements using validated weight-based algorithms, accounting for concurrent oral medications and insulin regimen intensity.
3. *Intervention module:* Simulates the introduction of GLP-1RAs and/or SGLT2is, incorporating meta-analytic data on medication effects and their interactions.
4. *Outcomes module:* Calculates health outcomes including severe hypoglycemia, cardiovascular events, kidney failure, weight changes, and mortality, translating these into DALYs.

Model parameters were derived from meta-analyses and observational cohorts (detailed in S1 Table).

### Data sources

We used the HPACC dataset, which includes nationally representative, individual-level cross-sectional surveys of diabetes and associated risk factors in LMICs conducted from 2005 onwards (see S1 Text). The dataset encompasses a wide range of demographic and socioeconomic variables, anthropometric and biological measures of disease status, and data on health service use. We have detailed each dataset’s survey methodology, sampling strategy, and timing in the Supporting information. Country-specific sampling weights were calculated using survey design information detailed in the Supporting information. When combining data across countries, we weighted each country’s contribution proportional to its population size while maintaining the survey design-based variance estimation structure. This approach preserves both within-country representativeness and appropriate weighting of countries’ relative contributions to the overall estimates. The survey data used in our assessment includes age, sex, measured body weight, hemoglobin A1c (%), whether the person was on diabetes medications, whether the person reported taking insulin, current tobacco smoking, blood pressure values, and blood pressure medication treatment, prior cardiovascular and cerebrovascular disease history, and lipid profile. Inclusion criteria for the analysis included current use of insulin and history of diabetes defined by self-reported diagnoses from a healthcare provider, fasting blood glucose 126 mg/dL (7 mmol/L) or higher, or hemoglobin A1c 6.5% or higher.

### Outcomes

The primary outcome of interest was reduction in insulin use per person per day if people with diabetes taking insulin started either an SGLT-2 inhibitor or GLP1-RA. The secondary outcome was total DALYs lost per 1,000-person years, adjusted for duration of effect, due to all causes that would be affected by choice of glycemic drugs, including severe hypoglycemia, cardiovascular disease (including atherosclerotic heart disease and stroke, as well as heart failure), kidney disease, weight change (other non-cardiorenal morbidity and mortality benefits of lower weight), and total all-cause mortality. The DALY calculation accounted for the morbidity and severity of each disease outcome, the timing of onset of each outcome, all-cause mortality by age and sex in each country, and the impact of treatment regimens on the rate of each outcome. We have detailed how these were calculated and modeled in the Supporting information. Additional outcomes included the fraction of DALYs lost to each specific cause.

### Analytic approach

A microsimulation model was employed to estimate the potential reduction in insulin use with the introduction of SGLT-2 inhibitors and GLP-1 receptor agonists. A microsimulation is a model that estimates changes in outcomes for individuals, rather than using population averages, to capture inequalities and distributions of outcomes across population sub-groups [20]. The model repeatedly samples from probability distributions of the input parameters (detailed below) to quantify the uncertainty and variability around the outcome metrics. The input parameters, modeling strategies, and several sensitivity analyses are detailed below, while the appendix provides a further detailed description of each step of the modeling. The complete R code implementing the microsimulation model is available at https://github.com/sanjaybasu/hpacc-insulin-glp1-sglt2 and https://doi.org/10.5281/zenodo.14634630.

#### Insulin dosage, change in dosage, and severe hypoglycemia rates.

Given that insulin dosage data were unavailable, the model used weight-based formulas to estimate a range of possible current insulin doses among individuals who reported using insulin, keeping any metformin they were currently using unchanged. Baseline insulin requirements were estimated using validated weight-based algorithms [21]. For the main analysis, we used a dosing factor of 0.64 IU/kg/day, representing typical requirements for patients on basal regimens. In sensitivity analyses, we modeled lower doses (0.37 IU/kg/day) representing basal-bolus regimens or patients on concurrent sulfonylureas, and higher doses (0.84 IU/kg/day) for more insulin-resistant patients. These ranges were derived from real-world insulin utilization studies in LMICs [22].

We simulated the reduction in insulin use after adding a GLP1-RA or SGLT2-i using randomized controlled trial data, assuming continued use of metformin when reducing insulin use and no addition of other medications beyond the GLP1-RA or SGLT2-i for the purposes of calculating the new insulin dosage. Specifically, for adding a GLP-1 receptor agonist, we used data from the SUSTAIN 5 trial, which indicated a 17% decrease in insulin units per person per day (95% CI: 14%, 19%) [23], while for adding an SGLT-2 inhibitor we used data from the EMPA-REG BASALTM, which indicated an 11% decrease in insulin units per person per day (95% CI: 6%, 16%) [24].

We also modeled the combined effects of simultaneous GLP-1RA and SGLT2i treatment based on recent meta-analyses [19,25–27]. Meta-analysis of randomized trials has shown that combination therapy provides additional benefits beyond monotherapy: further reduction in HbA1c (−0.74%), additional weight loss (−1.61 kg), and incremental systolic blood pressure reduction (−3.32 mmHg) compared to SGLT2i alone [25]. For our model, we incorporated these additive effects along with an incremental 20% reduction in insulin requirements beyond SGLT2i monotherapy (95% CI: 15%–25%) [19]. We modeled additional cardiovascular risk reduction (15% relative risk reduction) [26] while maintaining the favorable kidney outcomes profile of SGLT2i therapy. Side effect profiles were modeled as additive between the two drug classes, supported by safety data from combination therapy trials [27].

The microsimulation was implemented by:

1. Drawing parameter values from their respective distributions based on meta-analytic inputs;
2. Generating individual-level outcomes for the entire study population;
3. Calculating population-weighted estimates accounting for survey design; and
4. Storing results for uncertainty quantification.

We simulated the baseline rate of severe hypoglycemia events to be 5.2 per 100 patient-years in type 2 diabetes mellitus (T2DM, 95% CI: 4.2, 6.4) among people on insulin but not on a GLP1-RA or SGLT2-i [28], with a disutility of 0.10 (95% CI: 0.09, 0.11) for severe hypoglycemia events with a 1-day duration of disutility [29]. We used meta-analytic estimates to simulate the relative risk of severe hypoglycemia as 0.46 (95% CI: 0.38, 0.55) when adding a GLP1-RA [30] and 1.24 (95% CI: 0.77, 2.00) when adding an SGLT2-i [31].

Each of our individual outcome equations has been previously validated in published studies. The severe hypoglycemia risk equations were validated in international cohort studies [28], cardiovascular disease risk was validated through the Globorisk derivation datasets specific to each country [32], kidney disease progression was validated in longitudinal cohorts [33,34], and mortality equations were validated in national diabetes registries [35]. While these component equations were validated, we did not perform additional model calibration at the population level given the absence of true diabetes complication incidence data from most LMICs. We did address population-level competing risks through established mortality equations and incorporated parameter uncertainty through sampling from parameter distributions specified in S1 Table.

#### Adverse events from new medications.

We estimated the new major adverse event risks from GLP1-Ras and SGLT2-is. For GLP-1 receptor agonists, we included severe gastrointestinal side effects (15%–20% incidence with disutility 0.188 lasting 3–4 days [36]) and pancreatitis (1.2–2.1 events per 1,000 patient-years [37] with disutility 0.324 lasting 28–53 days [36]). For SGLT-2 inhibitors, we estimated the rate of urogenital infections (87.4 excess risk per 1,000 person-years for women and 11.9 for men [27]) and diabetic ketoacidosis (0.6–4.9 events per 1,000 person-years [38]), along with their respective disutilities (0.051 lasting 3–14 days [36], and 0.1–0.2 lasting 3–7 days [36]). We did not include the risk of amputation or fracture for SGLT2-i’s, given evidence that these severe adverse events were specific to canagliflozin and not a class effect [39,40].

#### Benefits from new medications.

We simulated the benefits of adding SGLT-2 inhibitors and GLP-1 receptor agonists, including reduced weight, cardiovascular disease, renal disease, and mortality. For GLP-1 receptor agonists, the analysis included a 3.4 kg weight loss (95% CI: 2.3–4.5 [25,41] with a disutility of 0.00012–0.00441 per kg [42]), an 18% reduction in major cardiovascular events (hazard ratio, 0.82; 95% CI, 0.68–0.98 [43], with a disutility varied from 0.041 to 0.179 [36], and a baseline cardiovascular event rate given by the Globorisk laboratory equation for each surveyed country [32]), a 21% reduction in kidney disease (hazard ratio, 0.79; 95% CI, 0.66–0.94 [43], with a disutility of 0.104–0.571 [36] from a baseline of 2.9–7.4 per 1,000 person-years from observational cohorts [33,34]), and a 20% reduction in all-cause mortality (hazard ratio, 0.80; 95% CI, 0.67–0.95 [43], with baseline mortality for people with diabetes modeled as 1.769 *  exp^(0.0565 *  age) per observational studies [35]). For SGLT-2 inhibitors, the analysis included a 1.8 kg weight loss (95% CI: 1.9–1.7) [25], a 15% reduction in major cardiovascular events (relative risk 0.85; 95% CI, 0.77–0.93) [44], a 37% reduction in the risk of kidney disease (relative risk 0.63; 95% CI, 0.58–0.69) [45], and a 21% reduction in all-cause mortality (relative risk 0.79; 95% CI, 0.70–0.88) [44], with the same disutilities for each condition.

We simulated the outcomes in terms of DALYs averted, by cause and overall, for each member of the total population in each country and overall, accounting for the fraction of each surveyed population who reported having a diagnosis of diabetes and taking insulin for diabetes. As some countries had a small number of people reporting insulin use, we limited our results for the individual country analyses to those countries with at least 100 participants in the survey reporting insulin use. We also reported the total among all countries regardless of sample size. We calculated DALYs per 1,000 person-years in each population at a standard 3% annual discount rate.

### Sensitivity and uncertainty analyses

We conducted several sensitivity analysis scenarios. We estimated lower and upper bounds of benefits in terms of reduced hypoglycemia risk using the lower baseline insulin use and higher baseline insulin use, reflecting alternative scenarios of basal plus bolus insulin or insulin use with sulfonylureas. We conducted sensitivity analyses to account for heterogeneity within drug classes. For GLP-1Ras, we separately modeled oral versus injectable formulations based on clinical guidance [46,47]. Oral formulations achieved roughly half the insulin-sparing effect of injectables modeled in the base case [48–50]. We performed uncertainty analysis by sampling from normal distributions constructed from the means and 95% confidence intervals around each input parameter to capture distributional effects (see S1 Table), generating uncertainty estimates around each outcome metric. We performed all modeling in R (version 4.4, The R Project for Statistical Computing, Vienna).

## Results

### Descriptive statistics

We included the most recent available dataset from each country in the analysis for a total of 79 countries sampling 4,837 people with diabetes using insulin across the time periods 2007–2020 (Table 1). Fourteen of the countries had >100 people reporting insulin use for diabetes. The median age of the insulin users was 57.0 years (IQR: 48.0, 64.0), and 62.3% were female. The median body weight was 75.0 kg (IQR: 64.0, 87.0), and the rate of insulin use in the overall population averaged 2.0% (ranging from 1.1% in Iran to 4.5% in Tonga, among those countries with *n* > 100 survey participants reporting insulin use).

**Table 1:** Baseline characteristics of insulin users in HPACC survey, showing overall among all countries regardless of sample size, and the subset of countries with *N* > 100 people with diabetes reporting insulin use.

	Overall	Algeria	Bangladesh	Brazil	Costa Rica	Egypt	Iran	Jordan	Libya	Marshall Islands	Mexico	Morocco	South Africa	Sudan	Tonga
n in HPACC survey	4837	172	109	629	131	227	359	157	131	106	606	108	246	106	173
Age, years, median [IQR]	57.00 [48.00, 64.00]	54.00 [42.75, 62.00]	50.00 [41.00, 58.00]	62.00 [52.00, 70.00]	61.50 [54.00, 72.00]	51.00 [43.50, 56.00]	60.00 [53.00, 67.50]	59.00 [51.00, 64.00]	52.00 [47.00, 60.00]	53.00 [47.00, 63.00]	60.00 [50.25, 69.00]	59.00 [51.00, 67.00]	57.00 [50.00, 66.00]	50.50 [41.00, 56.00]	53.00 [46.00, 61.00]
Female, *n* (%)	3011 (62.3)	110 (64.0)	52 (47.7)	395 (62.8)	96 (73.3)	153 (67.4)	227 (63.2)	98 (62.4)	71 (54.2)	60 (56.6)	364 (60.1)	78 (72.2)	158 (64.2)	68 (64.2)	115 (66.5)
Weight, kg, median [IQR]	75.00 [64.00, 87.40]	77.00 [68.10, 87.35]	64.00 [56.00, 70.25]	72.70 [63.10, 84.45]	73.50 [66.00, 85.00]	89.00 [76.00, 102.00]	75.00 [66.03, 83.47]	85.90 [74.93, 97.58]	83.00 [71.75, 92.80]	70.40 [62.30, 84.00]	73.35 [63.30, 83.73]	74.50 [65.00, 83.75]	76.50 [67.12, 90.62]	70.10 [62.85, 80.05]	95.35 [82.77, 106.05]
Estimated insulin, main case, IU/day, median [IQR]	48.00 [40.96, 55.94]	49.28 [43.58, 55.90]	40.96 [35.84, 44.96]	46.53 [40.38, 54.05]	47.04 [42.24, 54.40]	56.96 [48.64, 65.28]	48.00 [42.26, 53.42]	54.98 [47.95, 62.45]	53.12 [45.92, 59.39]	45.06 [39.87, 53.76]	46.94 [40.51, 53.59]	47.68 [41.60, 53.60]	48.96 [42.96, 58.00]	44.86 [40.22, 51.23]	61.02 [52.98, 67.87]
Estimated insulin, low case, IU/day, median [IQR]	27.75 [23.68, 32.34]	28.49 [25.20, 32.32]	23.68 [20.72, 25.99]	26.90 [23.35, 31.25]	27.20 [24.42, 31.45]	32.93 [28.12, 37.74]	27.75 [24.43, 30.89]	31.78 [27.72, 36.10]	30.71 [26.55, 34.34]	26.05 [23.05, 31.08]	27.14 [23.42, 30.98]	27.56 [24.05, 30.99]	28.30 [24.84, 33.53]	25.94 [23.25, 29.62]	35.28 [30.63, 39.24]
Estimated insulin, high case, IU/day, median [IQR]	63.00 [53.76, 73.42]	64.68 [57.20, 73.37]	53.76 [47.04, 59.01]	61.07 [53.00, 70.94]	61.74 [55.44, 71.40]	74.76 [63.84, 85.68]	63.00 [55.46, 70.12]	72.16 [62.94, 81.96]	69.72 [60.27, 77.95]	59.14 [52.33, 70.56]	61.61 [53.17, 70.33]	62.58 [54.60, 70.35]	64.26 [56.38, 76.12]	58.88 [52.79, 67.24]	80.09 [69.53, 89.08]
Percent of total population reporting both diabetes diagnosis and insulin use, %	1.96	2.47	1.33	1.08	3.92	1.49	1.18	2.80	3.78	3.55	3.30	1.99	3.04	1.37	4.50

### Insulin dosage, change in dosage, and severe hypoglycemia rates

The median estimated insulin dose was 48.0 IU/day (IQR: 41.0, 55.9), with low and high estimates of 27.8 IU/day (IQR: 23.7, 32.3) and 63.0 IU/day (IQR: 53.8, 73.4), respectively (Table 1). The median estimated insulin dose varied from 41.0 IU/day in Bangladesh to 61.0 IU/day in Tonga, among those countries with at least 100 survey participants reporting insulin use.

The median new insulin dose after adding a GLP-1 receptor agonist was 39.8 IU/day (IQR: 34.0, 46.4), with low and high estimates of 22.5 IU/day (IQR: 19.2, 26.2) and 54.2 IU/day (IQR: 46.2, 63.1), respectively (Table 2). The median reduction in insulin dose was 8.2 IU/day (IQR: 6.9, 9.5), which varied from a low of 7.0 IU/day in Bangladesh to 10.4 IU/day in Tonga.

**Table 2:** Estimated changes in insulin dosing with GLP-1 receptor agonist therapy, showing overall among all countries regardless of sample size, and the subset of countries with *N* > 100 people with diabetes reporting insulin use.

	Overall	Algeria	Bangladesh	Brazil	Costa Rica	Egypt	Iran	Jordan	Libya	Marshall Islands	Mexico	Morocco	South Africa	Sudan	Tonga
n in HPACC survey	4837	172	109	629	131	227	359	157	131	106	606	108	246	106	173
Estimated insulin after GLP1-RA, main case, IU/day, median [IQR]	39.84 [34.00, 46.43]	40.90 [36.17, 46.40]	34.00 [29.75, 37.32]	38.62 [33.52, 44.86]	39.04 [35.06, 45.15]	47.28 [40.37, 54.18]	39.84 [35.07, 44.34]	45.63 [39.80, 51.83]	44.09 [38.11, 49.30]	37.40 [33.09, 44.62]	38.96 [33.62, 44.48]	39.57 [34.53, 44.49]	40.64 [35.66, 48.14]	37.24 [33.39, 42.52]	50.65 [43.97, 56.33]
Estimated insulin after GLP1-RA, low case, IU/day, median [IQR]	22.48 [19.18, 26.19]	23.08 [20.41, 26.18]	19.18 [16.78, 21.05]	21.79 [18.91, 25.31]	22.03 [19.78, 25.47]	26.67 [22.78, 30.57]	22.48 [19.79, 25.02]	25.74 [22.46, 29.24]	24.88 [21.50, 27.81]	21.10 [18.67, 25.17]	21.98 [18.97, 25.09]	22.33 [19.48, 25.10]	22.93 [20.12, 27.16]	21.01 [18.84, 23.99]	28.58 [24.81, 31.78]
Estimated insulin after GLP1-RA, high case, IU/day, median [IQR]	54.18 [46.23, 63.14]	55.62 [49.20, 63.10]	46.23 [40.45, 50.75]	52.52 [45.58, 61.01]	53.10 [47.68, 61.40]	64.29 [54.90, 73.68]	54.18 [47.70, 60.30]	62.05 [54.13, 70.49]	59.96 [51.83, 67.04]	50.86 [45.01, 60.68]	52.99 [45.73, 60.49]	53.82 [46.96, 60.50]	55.26 [48.49, 65.47]	50.64 [45.40, 57.83]	68.88 [59.80, 76.61]
Change in estimated insulin after GLP1-RA, main case, IU/day, median [IQR]	8.16 [6.96, 9.51]	8.38 [7.41, 9.50]	6.96 [6.09, 7.64]	7.91 [6.87, 9.19]	8.00 [7.18, 9.25]	9.68 [8.27, 11.10]	8.16 [7.18, 9.08]	9.35 [8.15, 10.62]	9.03 [7.81, 10.10]	7.66 [6.78, 9.14]	7.98 [6.89, 9.11]	8.11 [7.07, 9.11]	8.32 [7.30, 9.86]	7.63 [6.84, 8.71]	10.37 [9.01, 11.54]
Change in estimated insulin after GLP1-RA, low case, IU/day, median [IQR]	5.27 [4.50, 6.14]	5.41 [4.79, 6.14]	4.50 [3.94, 4.94]	5.11 [4.44, 5.94]	5.17 [4.64, 5.98]	6.26 [5.34, 7.17]	5.27 [4.64, 5.87]	6.04 [5.27, 6.86]	5.83 [5.04, 6.52]	4.95 [4.38, 5.91]	5.16 [4.45, 5.89]	5.24 [4.57, 5.89]	5.38 [4.72, 6.37]	4.93 [4.42, 5.63]	6.70 [5.82, 7.46]
Change in estimated insulin after GLP1-RA, high case, IU/day, median [IQR]	8.82 [7.53, 10.28]	9.06 [8.01, 10.27]	7.53 [6.59, 8.26]	8.55 [7.42, 9.93]	8.64 [7.76, 10.00]	10.47 [8.94, 12.00]	8.82 [7.76, 9.82]	10.10 [8.81, 11.47]	9.76 [8.44, 10.91]	8.28 [7.33, 9.88]	8.63 [7.44, 9.85]	8.76 [7.64, 9.85]	9.00 [7.89, 10.66]	8.24 [7.39, 9.41]	11.21 [9.73, 12.47]

The median new insulin dose after adding an SGLT-2 inhibitor was 42.7 IU/day (IQR: 36.5, 49.8), with low and high estimates of 23.3 IU/day (IQR: 19.9, 27.2) and 59.2 IU/day (IQR: 50.5, 69.0), respectively (Table 3). The median reduction in insulin dose was 5.3 IU/day (IQR: 4.5, 6.2), which varied from a low of 4.5 IU/day in Bangladesh to 6.7 IU/day in Tonga.

**Table 3:** Estimated changes in insulin dosing with SGLT2 inhibitor therapy, showing overall among all countries regardless of sample size, and the subset of countries with *N* > 100 people with diabetes reporting insulin use.

	Overall	Algeria	Bangladesh	Brazil	Costa Rica	Egypt	Iran	Jordan	Libya	Marshall Islands	Mexico	Morocco	South Africa	Sudan	Tonga
n in HPACC survey	4837	172	109	629	131	227	359	157	131	106	606	108	246	106	173
Estimated insulin after SGLT-2i, main case, IU/day, median [IQR]	42.72 [36.45, 49.78]	43.86 [38.79, 49.75]	36.45 [31.90, 40.01]	41.41 [35.94, 48.10]	41.87 [37.59, 48.42]	50.69 [43.29, 58.10]	42.72 [37.61, 47.55]	48.93 [42.68, 55.58]	47.28 [40.87, 52.86]	40.10 [35.49, 47.85]	41.78 [36.06, 47.69]	42.44 [37.02, 47.70]	43.57 [38.23, 51.62]	39.93 [35.80, 45.60]	54.31 [47.15, 60.41]
Estimated insulin after SGLT-2i, low case, IU/day, median [IQR]	23.31 [19.89, 27.16]	23.93 [21.17, 27.15]	19.89 [17.40, 21.83]	22.60 [19.61, 26.25]	22.84 [20.51, 26.42]	27.66 [23.62, 31.70]	23.31 [20.52, 25.94]	26.70 [23.29, 30.33]	25.80 [22.30, 28.84]	21.88 [19.36, 26.11]	22.80 [19.67, 26.02]	23.15 [20.20, 26.03]	23.78 [20.86, 28.17]	21.79 [19.53, 24.88]	29.63 [25.73, 32.96]
Estimated insulin after SGLT-2i, high case, IU/day, median [IQR]	59.22 [50.53, 69.01]	60.80 [53.77, 68.97]	50.53 [44.22, 55.47]	57.40 [49.82, 66.68]	58.04 [52.11, 67.12]	70.27 [60.01, 80.54]	59.22 [52.13, 65.91]	67.83 [59.16, 77.05]	65.54 [56.65, 73.27]	55.59 [49.19, 66.33]	57.92 [49.98, 66.11]	58.83 [51.32, 66.13]	60.40 [53.00, 71.56]	55.35 [49.63, 63.21]	75.29 [65.36, 83.74]
Change in estimated insulin after SGLT-2i, main case, IU/day, median [IQR]	5.28 [4.51, 6.15]	5.42 [4.79, 6.15]	4.51 [3.94, 4.95]	5.12 [4.44, 5.95]	5.17 [4.65, 5.98]	6.27 [5.35, 7.18]	5.28 [4.65, 5.88]	6.05 [5.27, 6.87]	5.84 [5.05, 6.53]	4.96 [4.39, 5.91]	5.16 [4.46, 5.89]	5.24 [4.58, 5.90]	5.39 [4.73, 6.38]	4.94 [4.42, 5.64]	6.71 [5.83, 7.47]
Change in estimated insulin after SGLT-2i, low case, IU/day, median [IQR]	4.44 [3.79, 5.17]	4.56 [4.03, 5.17]	3.79 [3.32, 4.16]	4.30 [3.74, 5.00]	4.35 [3.91, 5.03]	5.27 [4.50, 6.04]	4.44 [3.91, 4.94]	5.09 [4.44, 5.78]	4.91 [4.25, 5.49]	4.17 [3.69, 4.97]	4.34 [3.75, 4.96]	4.41 [3.85, 4.96]	4.53 [3.97, 5.36]	4.15 [3.72, 4.74]	5.64 [4.90, 6.28]
Change in estimated insulin after SGLT-2i, high case, IU/day, median [IQR]	3.78 [3.23, 4.40]	3.88 [3.43, 4.40]	3.23 [2.82, 3.54]	3.66 [3.18, 4.26]	3.70 [3.33, 4.28]	4.49 [3.83, 5.14]	3.78 [3.33, 4.21]	4.33 [3.78, 4.92]	4.18 [3.62, 4.68]	3.55 [3.14, 4.23]	3.70 [3.19, 4.22]	3.75 [3.28, 4.22]	3.86 [3.38, 4.57]	3.53 [3.17, 4.03]	4.81 [4.17, 5.34]

### Disability-adjusted life years (DALYs)

Table 4 presents the expected changes in DALYs lost across scenarios per 1,000 person-years in the overall country population, accounting for the portion of the overall population taking insulin at the time of the survey, and the reduced frequency of severe hypoglycemia, cardiovascular disease, renal disease, and overall mortality in both GLP-1 receptor agonist and SGLT-2 inhibitor cases. We also accounted for the increased frequency of gastrointestinal side effects and pancreatitis (in the GLP-1 receptor agonist cases) and the increased frequency of urogenital infections and ketoacidosis (in the SGLT-2 inhibitor cases).

**Table 4:** Estimated impact on disability-adjusted life years (DALYs) of GLP1-RA or SGLT2-i treatment among people with diabetes taking insulin across treatment scenarios, showing overall among all countries regardless of sample size, and the subset of countries with *N* > 100 people with diabetes reporting insulin use.

	Overall	Algeria	Bangladesh	Brazil	Costa Rica	Egypt	Iran	Jordan	Libya	Marshall Islands	Mexico	Morocco	South Africa	Sudan	Tonga
n in HPACC survey	4837	172	109	629	131	227	359	157	131	106	606	108	246	106	173
DALYs, main case before GLP1-RA or SGLT2-i (median [IQR])	2.20 [1.49, 4.02]	2.70 [2.11, 3.49]	1.87 [1.41, 2.39]	1.64 [1.43, 1.93]	5.47 [4.33, 6.92]	1.97 [1.85, 2.09]	1.70 [1.39, 2.37]	3.74 [3.03, 4.43]	5.02 [4.44, 5.92]	3.94 [3.18, 5.16]	4.82 [4.07, 6.31]	2.61 [1.96, 3.43]	4.32 [3.99, 4.99]	1.92 [1.60, 2.34]	4.93 [3.87, 6.27]
DALYs, low case before GLP1-RA or SGLT2-i (median [IQR])	1.20 [0.76, 2.14]	1.40 [0.91, 1.98]	0.98 [0.65, 1.36]	0.95 [0.73, 1.22]	3.02 [2.25, 4.37]	1.00 [0.88, 1.12]	0.96 [0.67, 1.39]	2.06 [1.59, 2.60]	2.61 [2.14, 3.27]	2.03 [1.44, 2.95]	2.74 [2.01, 4.04]	1.45 [0.97, 2.07]	2.34 [2.01, 3.01]	0.97 [0.78, 1.27]	2.61 [1.78, 3.56]
DALYs, high case before GLP1-RA or SGLT2-i (median [IQR])	5.00 [3.55, 9.59]	6.24 [5.36, 7.91]	4.42 [3.52, 5.85]	3.71 [3.47, 4.01]	12.40 [10.12, 14.23]	4.81 [4.69, 4.93]	3.97 [3.18, 4.90]	8.76 [7.06, 9.70]	12.15 [10.71, 13.42]	9.40 [7.87, 11.67]	11.01 [10.33, 12.93]	6.02 [4.72, 7.31]	10.13 [9.81, 10.80]	4.63 [3.98, 5.62]	11.66 [9.60, 14.28]
DALYs, main case after GLP1-RA (median [IQR])	1.01 [0.61, 1.86]	1.01 [0.53, 1.65]	0.89 [0.51, 1.31]	0.81 [0.64, 1.05]	2.57 [1.63, 3.76]	0.90 [0.80, 0.99]	0.82 [0.56, 1.36]	1.69 [1.12, 2.25]	2.27 [1.81, 3.00]	1.49 [0.88, 2.49]	2.34 [1.74, 3.54]	1.17 [0.64, 1.83]	2.06 [1.80, 2.59]	0.90 [0.64, 1.25]	1.84 [0.98, 2.94]
DALYs, low case after GLP1-RA (median [IQR])	0.00 [0.00, 0.15]	0.00 [0.00, 0.00]	0.00 [0.00, 0.11]	0.01 [0.00, 0.27]	0.00 [0.00, 0.90]	0.00 [0.00, 0.00]	0.00 [0.00, 0.33]	0.00 [0.00, 0.15]	0.00 [0.00, 0.00]	0.00 [0.00, 0.00]	0.00 [0.00, 1.10]	0.00 [0.00, 0.31]	0.00 [0.00, 0.34]	0.00 [0.00, 0.02]	0.00 [0.00, 0.00]
DALYs, high case after GLP1-RA (median [IQR])	4.21 [3.02, 8.17]	5.35 [4.66, 6.71]	3.74 [3.03, 4.90]	3.12 [2.97, 3.32]	10.41 [8.58, 11.79]	4.10 [4.02, 4.17]	3.35 [2.72, 4.03]	7.51 [6.04, 8.12]	10.33 [9.18, 11.27]	8.03 [6.85, 9.89]	9.30 [8.85, 10.59]	5.07 [4.06, 6.13]	8.57 [8.35, 9.01]	3.91 [3.43, 4.71]	9.95 [8.33, 12.12]
DALYs, main case after SGLT-2i (median [IQR])	1.25 [0.81, 2.29]	1.35 [0.88, 2.01]	1.09 [0.71, 1.53]	0.97 [0.81, 1.21]	3.17 [2.20, 4.35]	1.12 [1.03, 1.21]	1.00 [0.73, 1.53]	2.11 [1.51, 2.67]	2.85 [2.38, 3.57]	2.01 [1.38, 3.01]	2.84 [2.24, 4.03]	1.46 [0.92, 2.13]	2.52 [2.26, 3.04]	1.11 [0.84, 1.47]	2.47 [1.61, 3.63]
DALYs, low case after SGLT-2i (median [IQR])	0.12 [0.00, 0.50]	0.00 [0.00, 0.35]	0.11 [0.00, 0.42]	0.22 [0.03, 0.46]	0.44 [0.00, 1.62]	0.04 [0.00, 0.14]	0.17 [0.00, 0.55]	0.23 [0.00, 0.70]	0.15 [0.00, 0.72]	0.00 [0.00, 0.58]	0.54 [0.00, 1.68]	0.15 [0.00, 0.69]	0.33 [0.05, 0.92]	0.08 [0.00, 0.32]	0.00 [0.00, 0.57]
DALYs, high case after SGLT-2i (median [IQR])	3.76 [2.67, 7.22]	4.54 [3.83, 5.96]	3.34 [2.60, 4.54]	2.80 [2.63, 3.00]	9.22 [7.32, 10.65]	3.64 [3.56, 3.72]	2.99 [2.34, 3.70]	6.65 [5.13, 7.29]	9.18 [7.99, 10.16]	6.89 [5.67, 8.82]	8.31 [7.84, 9.66]	4.45 [3.41, 5.55]	7.65 [7.43, 8.12]	3.50 [2.99, 4.32]	8.50 [6.82, 10.75]

The overall median DALYs lost per 1,000 person-years before the addition of GLP-1RAs or SGLT2-i’s was 2.20 (IQR: 1.49, 4.02). The median DALYs lost with the addition of GLP-1 receptor agonists were reduced to 1.01 (IQR: 0.61, 1.86). With SGLT-2 inhibitors, the median DALYs lost reduced to 1.25 (IQR: 0.81, 2.29).

When analyzing combination therapy with both GLP-1RA and SGLT2i (S2 Table), we found greater reductions in insulin requirements than with either therapy alone. The median insulin dose with combination therapy was 34.2 IU/day [IQR: 29.2, 39.8], representing an additional 20% reduction beyond SGLT2i monotherapy. DALYs lost were reduced to 1.1 [IQR: 0.6, 2.0] per 1,000 person-years with combination therapy. The greatest absolute benefits were seen in countries with higher baseline insulin utilization. In sensitivity analyses examining oral versus injectable GLP-1RAs, oral formulations achieved approximately half the insulin-sparing effect, with median insulin dose 43.2 IU/day [IQR: 36.9, 50.3].

In decomposition analyses examining sources of uncertainty, parameter uncertainty in the efficacy of newer medications contributed 47% of total outcome uncertainty, while uncertainty in baseline event rates contributed 31%, and sampling uncertainty contributed 22%. Results were robust across multiple sensitivity analyses varying distributional assumptions for parameter sampling (Table 4).

**Fig 1:**
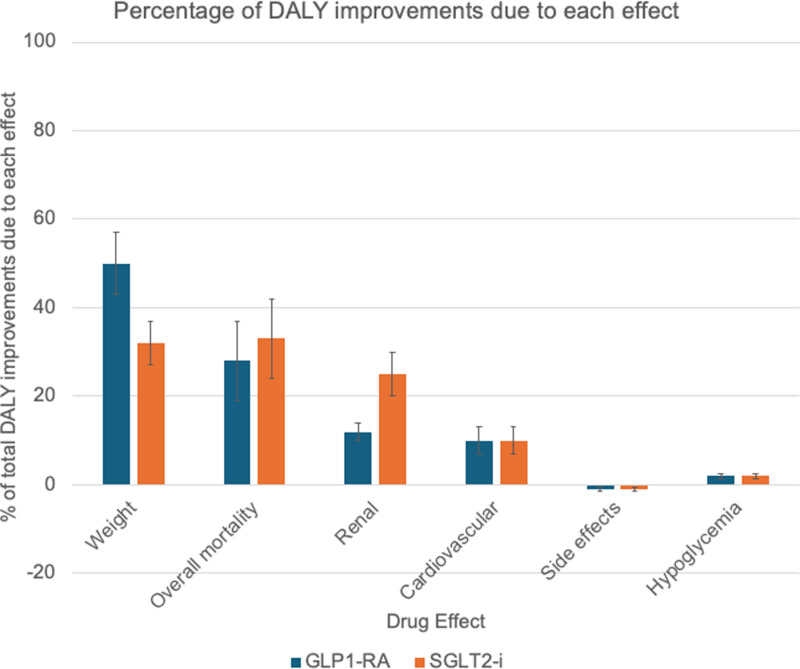
Relative contribution of different effects to overall improvement in disability-adjusted life years (DALYs) with GLP-1RA and SGLT2-i therapy. The chart shows the percentage contribution of each mechanism (hypoglycemia reduction, weight loss, cardiovascular protection, kidney protection, mortality reduction) to the total DALYs saved. Negative values for side effects indicate small quality-of-life decrements from medication adverse events. Error bars represent interquartile ranges across all countries in analysis.

Fig 1 illustrates the percentage contribution to DALYs gained per person with diabetes taking insulin of different effects from GLP-1 receptor agonist or SGLT-2 inhibitor medication use. The largest contributor to DALY improvements for both medication classes was the reduction in weight-related morbidity. For GLP-1 receptor agonists, weight reduction accounted for 50% (IQR: 43%, 57%) of the total improvement, while for SGLT-2 inhibitors, it accounted for 32% (IQR: 27%, 37%) of DALY improvements. The reduction in overall mortality was the second largest contributor, accounting for 28% (IQR: 19%, 36%) of improvement in GLP-1RA scenarios and 33% (IQR: 24%, 42%) of the total improvement in SGLT-2 inhibitor scenarios. Renal benefits contributed significantly, particularly for SGLT-2 inhibitors, accounting for 25% (IQR: 20%, 29%) of improvements, compared to 12% (IQR: 10%, 13%) for GLP-1 receptor agonists. Cardiovascular benefits contributed equally for both medication classes, accounting for 10% of improvements (IQR: 7%, 12% for GLP-1RAs; 7%, 13% for SGLT-2 inhibitors).

## Discussion

The results of this study highlight the potential benefits of introducing GLP-1 receptor agonists and SGLT-2 inhibitors for managing type 2 diabetes mellitus in LMICs. By reducing the burden of excess weight, cardiovascular and renal morbidity, and overall mortality, these novel therapies could significantly improve health outcomes and reduce disability among individuals with diabetes who are currently reliant on insulin—particularly in the case of GLP-1 receptor agonists. In previous work, we studied cost reductions needed for such therapies to be more widely accessible in LMICs [15]. We also note that newer GLP-1 receptor agonists that are oral rather than injected will affect pricing, refrigeration, and availability in coming years [16]. But access to GLP-1 receptor agonists and SGLT-2 inhibitors has been dramatically increasing in recent years, particularly given the presence of generics (e.g., for dapagliflozin and liraglutide) and compounded formulations [14,17]. The microsimulation model employed in this study demonstrates that the addition of GLP-1 receptor agonists in particular, but also SGLT-2 inhibitors, could lead to a substantial reduction in insulin dosage, with median reductions of 8.2 IU/day and 5.3 IU/day, respectively. This decrease in insulin use is particularly important in LMICs, where access to consistent insulin supply, refrigeration, and glucose monitoring devices and test strips remains challenging.

Our analysis of combination therapy with both GLP-1RA and SGLT2i showed greater benefits than either therapy alone, consistent with recent meta-analyses showing additive effects on glycemic control, weight loss, and cardiovascular risk reduction [19,25–27]. The additional 20% reduction in insulin requirements with combination therapy could have particular importance in settings where insulin access and storage are challenging. However, we note that cost considerations may limit widespread adoption of dual therapy in many LMICs. Our sensitivity analyses examining oral versus injectable GLP-1RAs demonstrated more modest insulin-sparing effects with oral formulations, though these agents may offer advantages in settings where injectable medication storage and administration are barriers to care.

In addition to the benefits of the newer medications for reducing severe hypoglycemia, as well as cardiovascular and renal morbidity and mortality, our study also accounted for the potential side effects associated with GLP-1 receptor agonists and SGLT-2 inhibitors, such as gastrointestinal issues, pancreatitis, urogenital infections, and ketoacidosis. However, the impact of these side effects on DALYs was minimal at the population level, suggesting that the benefits of these therapies likely outweigh the risks in most cases.

The results of this study provide valuable evidence for policymakers and healthcare providers in LMICs who are increasingly incorporating GLP-1 receptor agonists and SGLT-2 inhibitors into diabetes management protocols. By demonstrating the potential benefits of these therapies in reducing insulin dosage, severe hypoglycemia, cardiovascular and renal morbidity, and overall disability and mortality, this study supports the case for increasing access to these medications in LMICs, particularly GLP-1 receptor agonists, given our finding of particularly strong benefits in terms of DALYs for this medication class. Beyond the clinical implications of the shift between insulin and GLP-1 receptor agonists and SGLT-2 inhibitors, a broader market view is needed in terms of what will happen to the insulin market. As two of the biggest three insulin producers are also key players in the GLP-1 receptor agonists market, production capacity may be shifted from insulin to produce newer, more profitable medicines. Such market forces may have wider impacts on the availability and affordability of insulin for both type 1 and type 2 diabetes.

While considering these uncertainties, it is important to acknowledge the limitations of our current study. The data informing our model do not differentiate between type 1 and type 2 diabetes. Although over 95% of people with diabetes in the studied age range in LMICs are considered type 2 [3], the benefits of the medications under study would not extend to those with type 1. Additionally, data do not distinguish between prandial versus basal insulin, and typically most hypoglycemia with insulin will occur in association with prandial insulin. Our microsimulation model relies on assumptions and estimates derived from randomized trials and observational cohorts, which may not fully capture the real-world effectiveness of these therapies in LMICs, and not fully consider extreme outliers and rare events. Additionally, our study did not consider the cost-effectiveness of these therapies, given limited systematic data on pricing across LMICs, which is a critical factor in decision-making for resource-limited settings. Multiple small molecule oral GLP-1 agonists that seem highly effective and are in the pipeline; these drugs could arguably be made at lower cost and would not need a cold chain [16]. Future research should focus on conducting real-world studies in LMICs to assess the cost-effectiveness of GLP-1 receptor agonists and SGLT-2 inhibitors in these settings; we have applied our models to theoretical cost-effectiveness analyses of these medicines previously [17]. We did not evaluate the complete discontinuation of insulin therapy or possibly delaying or avoiding the need to start insulin in some individuals in the context of limited data. We also did not examine the potential for worsening retinopathy with high-dose GLP-1RAs in the first year after rapid glucose reduction in light of continued uncertainties about whether and to what degree such risk exists [29,30]. We also acknowledge that the effects of combination therapy may vary across populations and healthcare settings in ways not captured by our model. Additionally, while we examined differences between oral and injectable GLP-1RAs, real-world effectiveness may differ from trial-based estimates, particularly in resource-limited settings where medication adherence and monitoring may be suboptimal.

In conclusion, our current study provides compelling evidence for the potential benefits of introducing GLP-1 receptor agonists and SGLT-2 inhibitors for managing type 2 diabetes mellitus in LMICs. By reducing insulin dosage and the associated risk of severe hypoglycemia, as well as cardiovascular and renal morbidity and mortality, these therapies could significantly improve health outcomes and reduce disability among individuals with diabetes. Policymakers and healthcare providers should consider these findings when making decisions about the allocation of resources and the development of diabetes management strategies in LMICs. Further research is needed to validate these results in real-world settings to assess the effectiveness of these therapies in resource-limited contexts.

## Supporting information

S1 Fig Structure of microsimulation model for evaluating impact of GLP-1 receptor agonists and SGLT2 inhibitors. (DOCX)

S1 Table Input parameters to the microsimulation model. Uncertainty intervals were estimated through Monte Carlo sampling with replacement from the 95% confidence intervals around each input parameter using a Gaussian distribution around the mean to generate the confidence intervals around the outcome.
(DOCX)

S2 Table Impact of combination therapy and oral GLP-1 receptor agonist formulations on insulin dosing and health outcomes. Results shown by country for combination therapy with both GLP-1 receptor agonist and SGLT2 inhibitor (“combo”) and for oral GLP-1 receptor agonist monotherapy (“oral”). Insulin doses are in international units (IU) per day. DALYs represent disability-adjusted life years lost per 1,000 person-years.
(DOCX)

S1 Text Microsimulation model overview. (DOCX)

## Abbreviations

DALYs: disability-adjusted life years
GLP-1: glucagon-like peptide 1
HPACC: Global Health and Population Project on Access to Care for Cardiometabolic Diseases
LMICs: low- and middle-income countries
SGLT-2: sodium-glucose co-transporter-2
