# Supplementary material for: Reduced insulin use and diabetes complications upon introduction of SGLT-2 inhibitors and GLP1-receptor agonists in low- and middle-income countries: A microsimulation
Source: PLoS Med. 2025 Apr 17;22(4):e1004559. doi: 10.1371/journal.pmed.1004559 (PMC12005516; doi:10.1371/journal.pmed.1004559)
Supplement: S1 Fig — (DOCX) [file pmed.1004559.s001.docx]

***S1 Figure***: Structure of microsimulation model for evaluating impact of GLP-1 receptor agonists and SGLT2 inhibitors.

The model consists of four primary modules (shown in colored boxes). The Population Module generates synthetic individuals based on HPACC survey data, incorporating demographics, clinical parameters, and country-specific factors. The Insulin Dosage Module calculates baseline insulin requirements using validated weight-based algorithms that account for concurrent medications. The Intervention Module simulates the introduction of GLP-1 receptor agonists and/or SGLT2 inhibitors, alone or in combination. The Outcomes Module calculates health impacts including severe hypoglycemia, cardiovascular disease events, kidney disease progression, and resulting disability-adjusted life years (DALYs). Arrows indicate the flow of information between modules.

***
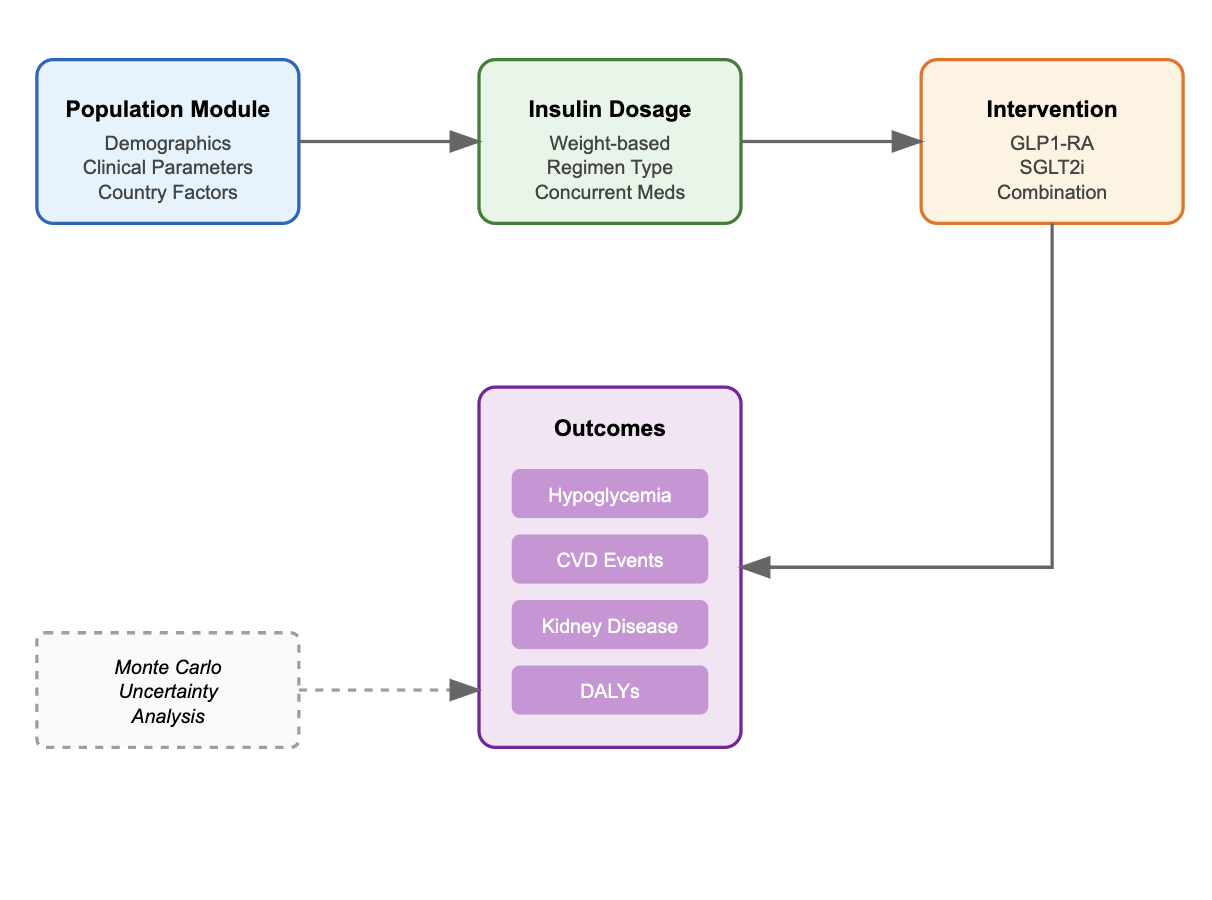
***
