# Supplementary material for: Reduced insulin use and diabetes complications upon introduction of SGLT-2 inhibitors and GLP1-receptor agonists in low- and middle-income countries: A microsimulation
Source: PLoS Med. 2025 Apr 17;22(4):e1004559. doi: 10.1371/journal.pmed.1004559 (PMC12005516; doi:10.1371/journal.pmed.1004559)
Supplement: S1 Table — Uncertainty intervals were estimated through Monte Carlo sampling with replacement from the 95% confidence intervals around each input parameter using a Gaussian distribution around the mean to generate the confidence intervals around the outcome. (DOCX) [file pmed.1004559.s002.docx]

***S1 Table***: Input parameters to the microsimulation model. Uncertainty intervals were estimated through Monte Carlo sampling with replacement from the 95% confidence intervals around each input parameter using a Gaussian distribution around the mean to generate the confidence intervals around the outcome.

| **Parameter** | **Mean** | **95% CI** | **Citation (see Supplemental References list)** |
| --- | --- | --- | --- |
| Insulin dosage (IU/kg/day) | 0.64 | [0.37, 0.84] | 1 |
| GLP-1 RA insulin reduction (%) | 17 | [14, 19] | 2 |
| SGLT-2i insulin reduction (%) | 11 | [6, 16] | 3 |
| Baseline severe hypoglycemia rate (per 100 patient-years) | 5.2 | [4.2, 6.4] | 4 |
| Severe hypoglycemia disutility | 0.10 | [0.09, 0.11] | 5 |
| GLP-1 RA relative risk of severe hypoglycemia | 0.46 | [0.38, 0.55] | 6 |
| SGLT-2i relative risk of severe hypoglycemia | 1.24 | [0.77, 2.00] | 7 |
| GLP-1 RA severe GI side effects (%) | 17.5 | [15, 20] | 8 |
| GLP-1 RA pancreatitis rate (per 1000 patient-years) | 1.65 | [1.2, 2.1] | 9, 10 |
| GI side effects disutility | 0.188 | [0.161, 0.215] | 11, 12 |
| Pancreatitis disutility | 0.324 | [0.224, 0.424] | 11, 13 |
| SGLT-2i urogenital infection rate (per 1000 person-years, women) | 87.4 | [30.8, 144.0] | 14 |
| SGLT-2i urogenital infection rate (per 1000 person-years, men) | 11.9 | [7.1, 16.7] | 14 |
| SGLT-2i diabetic ketoacidosis rate (per 1000 person-years) | 2.75 | [0.6, 4.9] | 15, 16 |
| Urogenital infection disutility | 0.051 | [0.06, 0.289] | 11, 17 |
| Diabetic ketoacidosis disutility | 0.15 | [0.1, 0.2] | 11, 18 |
| GLP-1 RA weight loss (kg) | 3.4 | [2.3, 4.5] | 19, 20 |
| SGLT-2i weight loss (kg) | 1.8 | [1.7, 1.9] | 19 |
| Weight change disutility (per kg) | 0.00185 | [0.00012, 0.00441] | 21 |
| GLP-1 RA major CV events hazard ratio | 0.82 | [0.68, 0.98] | 22 |
| GLP-1 RA end-stage renal disease hazard ratio | 0.79 | [0.66, 0.94] | 22 |
| GLP-1 RA all-cause mortality hazard ratio | 0.80 | [0.67, 0.95] | 22 |
| SGLT-2i major CV events relative risk | 0.85 | [0.77, 0.93] | 23 |
| SGLT-2i end-stage renal disease relative risk | 0.63 | [0.58, 0.69] | 24 |
| SGLT-2i all-cause mortality relative risk | 0.79 | [0.70, 0.88] | 23 |
| Cardiovascular event disutility | 0.072 | [0.041, 0.179] | 11 |
| End-stage renal disease disutility | 0.338 | [0.104, 0.571] | 11 |
| End-stage renal disease rate (per 1000 person-years) | 4.1 | [2.9, 7.4] | 25, 26 |
