# Supplementary material for: Reduced insulin use and diabetes complications upon introduction of SGLT-2 inhibitors and GLP1-receptor agonists in low- and middle-income countries: A microsimulation
Source: PLoS Med. 2025 Apr 17;22(4):e1004559. doi: 10.1371/journal.pmed.1004559 (PMC12005516; doi:10.1371/journal.pmed.1004559)
Supplement: S2 Table — Results shown by country for combination therapy with both GLP-1 receptor agonist and SGLT2 inhibitor (“combo”) and for oral GLP-1 receptor agonist monotherapy (“oral”). Insulin doses are in international units (IU) per day. DALYs represent disability-adjusted life years lost per 1,000 person-years. (DOCX) [file pmed.1004559.s003.docx]

***S2 Table:*** Impact of combination therapy and oral GLP-1 receptor agonist formulations on insulin dosing and health outcomes. Results shown by country for combination therapy with both GLP-1 receptor agonist and SGLT2 inhibitor ("combo") and for oral GLP-1 receptor agonist monotherapy ("oral"). Insulin doses are in international units (IU) per day. DALYs represent disability-adjusted life years lost per 1000 person-years.

|  | Overall | Algeria | Bangladesh | Brazil | Costa Rica | Egypt | Iran | Jordan | Libya | Marshall Islands | Mexico | Morocco | South Africa | Sudan | Tonga |
| --- | --- | --- | --- | --- | --- | --- | --- | --- | --- | --- | --- | --- | --- | --- | --- |
| n | 4837 | 172 | 109 | 629 | 131 | 227 | 359 | 157 | 131 | 106 | 606 | 108 | 246 | 106 | 173 |
| Daily insulin dose with combination therapy, IU/day (median [IQR]) | 34.18 [29.16, 39.83] | 35.09 [31.03, 39.80] | 29.16 [25.52, 32.01] | 33.13 [28.75, 38.48] | 33.49 [30.07, 38.73] | 40.56 [34.63, 46.48] | 34.18 [30.09, 38.04] | 39.14 [34.14, 44.46] | 37.82 [32.70, 42.29] | 32.08 [28.39, 38.28] | 33.42 [28.84, 38.15] | 33.95 [29.62, 38.16] | 34.86 [30.59, 41.30] | 31.94 [28.64, 36.48] | 43.45 [37.72, 48.32] |
| Daily insulin dose with combination therapy - low estimate, IU/day (median [IQR]) | 16.32 [13.92, 19.01] | 16.75 [14.82, 19.00] | 13.92 [12.18, 15.28] | 15.82 [13.73, 18.37] | 15.99 [14.36, 18.49] | 19.36 [16.53, 22.19] | 16.32 [14.36, 18.16] | 18.69 [16.30, 21.23] | 18.06 [15.61, 20.19] | 15.32 [13.55, 18.28] | 15.96 [13.77, 18.22] | 16.21 [14.14, 18.22] | 16.64 [14.60, 19.72] | 15.25 [13.67, 17.42] | 20.74 [18.01, 23.07] |
| Daily insulin dose with combination therapy - high estimate, IU/day (median [IQR]) | 53.30 [45.48, 62.11] | 54.72 [48.39, 62.07] | 45.48 [39.80, 49.92] | 51.66 [44.84, 60.01] | 52.23 [46.90, 60.40] | 63.25 [54.01, 72.49] | 53.30 [46.92, 59.32] | 61.04 [53.24, 69.34] | 58.98 [50.99, 65.95] | 50.03 [44.27, 59.69] | 52.13 [44.98, 59.50] | 52.94 [46.19, 59.52] | 54.36 [47.70, 64.40] | 49.82 [44.66, 56.89] | 67.76 [58.82, 75.36] |
| DALYs lost with combination therapy, per 1000 person-years (median [IQR]) | 1.07 [0.64, 2.00] | 1.00 [0.51, 1.77] | 0.96 [0.54, 1.47] | 0.87 [0.69, 1.10] | 2.74 [1.68, 3.92] | 0.98 [0.88, 1.07] | 0.90 [0.58, 1.45] | 1.78 [1.12, 2.40] | 2.45 [1.97, 3.23] | 1.56 [0.85, 2.67] | 2.52 [1.91, 3.75] | 1.21 [0.64, 1.94] | 2.23 [1.97, 2.75] | 0.98 [0.68, 1.41] | 1.93 [0.93, 3.18] |
| DALYs lost with combination therapy - low estimate, per 1000 person-years (median [IQR]) | 0.12 [0.00, 0.50] | 0.00 [0.00, 0.35] | 0.11 [0.00, 0.42] | 0.22 [0.03, 0.46] | 0.44 [0.00, 1.62] | 0.04 [0.00, 0.14] | 0.17 [0.00, 0.55] | 0.23 [0.00, 0.70] | 0.15 [0.00, 0.72] | 0.00 [0.00, 0.58] | 0.54 [0.00, 1.68] | 0.15 [0.00, 0.69] | 0.33 [0.05, 0.92] | 0.08 [0.00, 0.32] | 0.00 [0.00, 0.57] |
| DALYs lost with combination therapy - high estimate, per 1000 person-years (median [IQR]) | 3.76 [2.67, 7.22] | 4.54 [3.83, 5.96] | 3.34 [2.60, 4.54] | 2.80 [2.63, 3.00] | 9.22 [7.32, 10.65] | 3.64 [3.56, 3.72] | 2.99 [2.34, 3.70] | 6.65 [5.13, 7.29] | 9.18 [7.99, 10.16] | 6.89 [5.67, 8.82] | 8.31 [7.84, 9.66] | 4.45 [3.41, 5.55] | 7.65 [7.43, 8.12] | 3.50 [2.99, 4.32] | 8.50 [6.82, 10.75] |
| Daily insulin dose with oral GLP-1 receptor agonist, IU/day (median [IQR]) | 43.20 [36.86, 50.34] | 44.35 [39.23, 50.31] | 36.86 [32.26, 40.46] | 41.88 [36.35, 48.64] | 42.34 [38.02, 48.96] | 51.26 [43.78, 58.75] | 43.20 [38.03, 48.08] | 49.48 [43.16, 56.20] | 47.81 [41.33, 53.45] | 40.55 [35.88, 48.38] | 42.25 [36.46, 48.23] | 42.91 [37.44, 48.24] | 44.06 [38.66, 52.20] | 40.38 [36.20, 46.11] | 54.92 [47.68, 61.08] |
