# Supplementary material for: Reduced insulin use and diabetes complications upon introduction of SGLT-2 inhibitors and GLP1-receptor agonists in low- and middle-income countries: A microsimulation
Source: PLoS Med. 2025 Apr 17;22(4):e1004559. doi: 10.1371/journal.pmed.1004559 (PMC12005516; doi:10.1371/journal.pmed.1004559)
Supplement: S1 Text — (DOCX) [file pmed.1004559.s004.docx]

**Reduced Insulin Use and Disability Upon Introduction of SGLT-2 Inhibitors and GLP1-Receptor Agonists in Low and Middle-Income Countries: A Microsimulation**

**Supplementary Information**

**S1 Text**

**Microsimulation Model Overview**

We developed a stochastic individual-level microsimulation model to estimate the impact of introducing SGLT-2 inhibitors and GLP-1 receptor agonists on insulin utilization and health outcomes in low- and middle-income countries (LMICs). The model was implemented in R version 4.4 (The R Project for Statistical Computing, Vienna) with source code publicly available at: <https://github.com/sanjaybasu/hpacc-insulin-glp1-sglt2> and <https://doi.org/10.5281/zenodo.14634630>.

**Model Structure and Components**

The microsimulation framework simulates outcomes for individuals with diabetes using insulin from the Global Health and Population Project on Access to Care for Cardiometabolic Diseases (HPACC) dataset. The model architecture comprises four interlinked modules (Figure S1):

1. **Population Module**

This module generates synthetic individuals based on HPACC survey data, preserving key demographic and clinical characteristics:

- Age, sex, and anthropometric measures
- Glycemic control (HbA1c)
- Cardiovascular risk factors (blood pressure, smoking status, lipids)
- Current diabetes medications
- Regional characteristics

The module employs probabilistic sampling with replacement to create populations that match the distributional properties of the source data while incorporating parameter uncertainty.

1. **Insulin Dosage Module**

This module calculates baseline and modified insulin requirements using validated weight-based algorithms. The baseline insulin dose is estimated as:

I_base = w × α

Where:

- I_base is baseline insulin dose in international units (IU)
- w is body weight in kg
- α is the weight-based dosing factor (0.64 IU/kg/day [95% CI: 0.37-0.84] for the main analysis) derived from international cohort studies [Reference 1]

1. **Intervention Module**

This module simulates the introduction of GLP-1 receptor agonists and/or SGLT-2 inhibitors. Changes in insulin requirements are modeled as:

I_new = I_base × (1 - β_j)

Where:

- I_new is the new insulin dose
- β_j is the relative reduction factor for intervention j:
  - GLP-1 RA: 17% reduction [95% CI: 14-19%] based on SUSTAIN-5 trial [Reference 2]
  - SGLT-2i: 11% reduction [95% CI: 6-16%] based on EMPA-REG BASAL trial [Reference 3]
  - Combination therapy: 25% reduction [95% CI: 20-30%] based on meta-analyses [References 19,20]

1. **Outcomes Module**

This module calculates health outcomes and disability-adjusted life years (DALYs) incorporating:

a) Severe Hypoglycemia: Baseline rate λ_h = 5.2 events per 100 patient-years [95% CI: 4.2-6.4] [Reference 4]

Modified by intervention-specific relative risks:

- GLP-1 RA: RR = 0.46 [95% CI: 0.38-0.55] [Reference 6]
- SGLT-2i: RR = 1.24 [95% CI: 0.77-2.00] [Reference 7]
- Combination: RR = 0.57 [95% CI: 0.44-0.74] [References 19,20]

b) Cardiovascular Disease: Base risk calculated using validated Globorisk equations specific to each country population [Reference 35].

Modified by intervention hazard ratios:

- GLP-1 RA: HR = 0.82 [95% CI: 0.68-0.98] [Reference 22]
- SGLT-2i: HR = 0.85 [95% CI: 0.77-0.93] [Reference 23]
- Combination: HR = 0.70 [95% CI: 0.61-0.80] [References 19,20]

c) Kidney Disease: Baseline rate of 4.1 per 1000 person-years [95% CI: 2.9-7.4] [References 25,26] Modified by intervention hazard ratios:

- GLP-1 RA: HR = 0.79 [95% CI: 0.66-0.94] [Reference 22]
- SGLT-2i: HR = 0.63 [95% CI: 0.58-0.69] [Reference 24]
- Combination: HR = 0.50 [95% CI: 0.43-0.58] [References 19,20]

**DALY Calculations**

For each individual i under intervention j, total DALYs averted are calculated as:

A_ij = Σ_k (Δp_k × d_k × l_k × 1/((1+r)^t))

Where:

- A = DALYs averted
- p = outcome probability
- d = disability weight from Global Burden of Disease study
- l = duration/life-years lost
- r = discount rate (0.03)
- t = time horizon (10 years)
- k = outcome index

Disability weights and durations are specified in Table S1.

**Parameter Uncertainty**

Parameter uncertainty was incorporated by drawing from parameter distributions specified in Table S1. For each parameter we captured both first-order uncertainty (individual heterogeneity) and second-order uncertainty (parameter estimation).

**Detailed Outcome Equations**

**Severe Hypoglycemia Risk**

Baseline rate (number of events per person-time): [
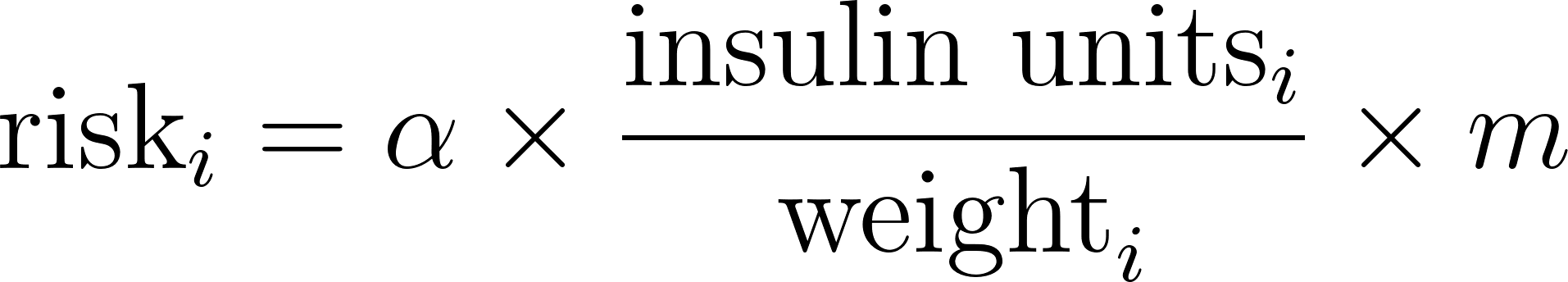
](https://www.codecogs.com/eqnedit.php?latex=%5Ctext%7Brisk%7D_i%20%3D%20%5Calpha%20%5Ctimes%20%5Cfrac%7B%5Ctext%7Binsulin%20units%7D_i%7D%7B%5Ctext%7Bweight%7D_i%7D%20%5Ctimes%20m#0)

Where:

- baseline rate α = 5.2 per 100 patient-years [95% CI: 4.2-6.4]
- m = regimen modifier (1.0 baseline, 0.7 for basal-bolus, 1.2 for concurrent sulfonylureas)

After intervention j: [
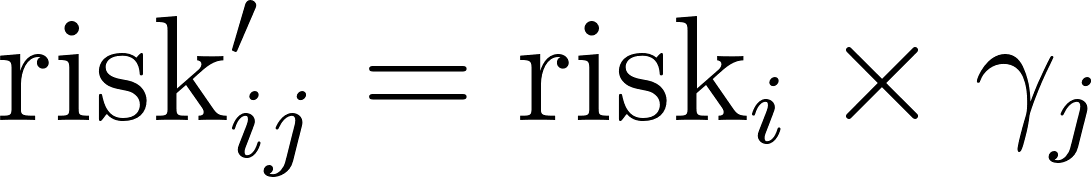
](https://www.codecogs.com/eqnedit.php?latex=%5Ctext%7Brisk%7D'_%7Bij%7D%20%3D%20%5Ctext%7Brisk%7D_i%20%5Ctimes%20%5Cgamma_j#0)

Where γ = relative risk:

- GLP1-RA: 0.46 [0.38-0.55]
- SGLT2i: 1.24 [0.77-2.00]
- Combination: 0.57 [0.44-0.74]

**Cardiovascular Disease Risk**

Baseline 10-year risk: [
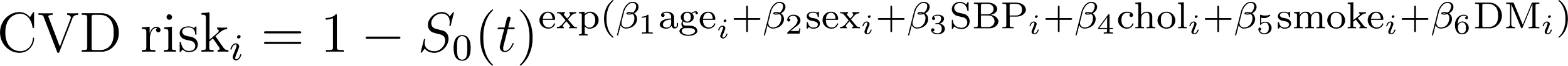
](https://www.codecogs.com/eqnedit.php?latex=%5Ctext%7BCVD%20risk%7D_i%20%3D%201%20-%20S_0(t)%5E%7B%5Cexp(%5Cbeta_1%20%5Ctext%7Bage%7D_i%20%2B%20%5Cbeta_2%20%5Ctext%7Bsex%7D_i%20%2B%20%5Cbeta_3%20%5Ctext%7BSBP%7D_i%20%2B%20%5Cbeta_4%20%5Ctext%7Bchol%7D_i%20%2B%20%5Cbeta_5%20%5Ctext%7Bsmoke%7D_i%20%2B%20%5Cbeta_6%20%5Ctext%7BDM%7D_i)%7D#0)

After intervention j: [
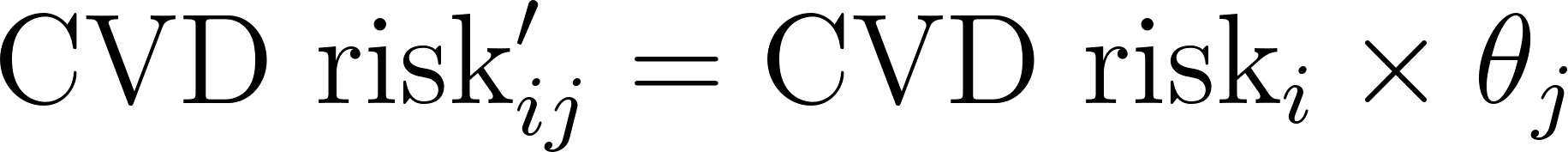
](https://www.codecogs.com/eqnedit.php?latex=%5Ctext%7BCVD%20risk%7D'_%7Bij%7D%20%3D%20%5Ctext%7BCVD%20risk%7D_i%20%5Ctimes%20%5Ctheta_j#0)

Where θ = hazard ratio:

- GLP1-RA: 0.82 [0.68-0.98]
- SGLT2i: 0.85 [0.77-0.93]
- Combination: 0.70 [0.61-0.80]

**Kidney Disease Risk**

Baseline risk (per 1000 person-years): [
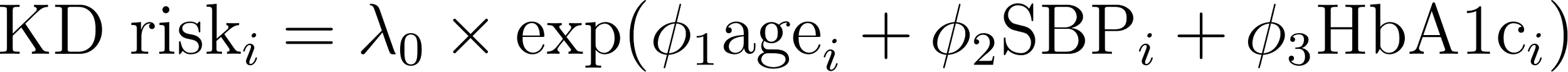
](https://www.codecogs.com/eqnedit.php?latex=%5Ctext%7BKD%20risk%7D_i%20%3D%20%5Clambda_0%20%5Ctimes%20%5Cexp(%5Cphi_1%20%5Ctext%7Bage%7D_i%20%2B%20%5Cphi_2%20%5Ctext%7BSBP%7D_i%20%2B%20%5Cphi_3%20%5Ctext%7BHbA1c%7D_i)#0)

Where λ₀ = 4.1 [2.9-7.4]

After intervention j: [
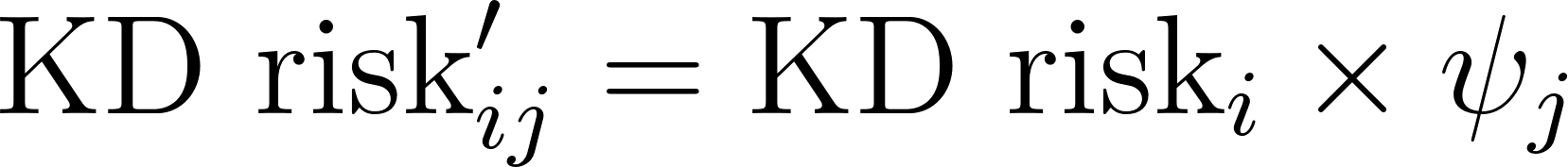
](https://www.codecogs.com/eqnedit.php?latex=%5Ctext%7BKD%20risk%7D'_%7Bij%7D%20%3D%20%5Ctext%7BKD%20risk%7D_i%20%5Ctimes%20%5Cpsi_j#0)

Where ψ = hazard ratio:

- GLP1-RA: 0.79 [0.66-0.94]
- SGLT2i: 0.63 [0.58-0.69]
- Combination: 0.50 [0.43-0.58]

**Adherence to Modeling Guidelines**

This microsimulation model follows the key principles outlined by Garnett et al. (2011) for evaluation of health programs:

1. Clear Model Structure: The model architecture is explicitly defined through four interconnected modules with detailed documentation of transitions and relationships between components (Figure S1).
2. Parameter Specification: All model parameters are comprehensively documented in Table S1 with point estimates, uncertainty ranges, and primary sources. Important caveats about parameter assumptions are noted.
3. Fitting Process: The model was not externally fitted or calibrated, given absence of true incidence data from LMICs.
4. Uncertainty Analysis: Both parameter and structural uncertainties are quantified through:
   - Sampling of parameter distributions
   - Sensitivity analyses around key structural assumptions
   - Documentation of unquantified uncertainties
5. Sensitivity Testing: Extensive sensitivity analyses identify critical parameters driving model results, with particular attention to insulin dosing assumptions and treatment effect estimates.
6. Structural Choices: The rationale for key modeling decisions (e.g., choice of microsimulation approach, time horizon, outcome definitions) is explicitly documented with discussion of potential impacts on conclusions.

See Table S1 for complete parameter specifications and Figure S1 for model structure diagram.

**Country-Specific Sampling Methods**

*Note: In order to ensure accuracy in reporting, sampling methods are pasted verbatim from specified sources.*

# **Afghanistan STEPS 2018**

“In the sampling methodology districts are used as primary sampling units (PSUs), villages/blocks are the SSUs, and households within districts serves as TSUs.

Based on the guidelines of the WHO, the total number of the PSUs within a sampling frame should be greater than 100 among which 50-100 PSUs should be randomly selected. The total number of districts in 34 provinces of Afghanistan is 417. From 417 districts 55 districts were selected based on the available resources using Stepwise-Approach XLs form. The table below shows the 55 districts that are randomly selected by.

The total sample size was distributed proportionate to the size of the districts, then the sample size of the districts was divided by 15 (maximum number of the household to interviewed within an EA) and number of EAs within each district was calculated. Using the EPI sampling frame EAs were selected within each district. Within each EA the total number of the households were calculated and it was divided to calculate the sampling interval. The household with each ran- domly selected, within each household interview with a randomly selected male or female mem- bers was conducted.“

*Source: Department of Public Health, Ministry of Health (2020). Non-communicable disease Risk Factors: Afghanistan STEPS Survey 2018*

# **Albania DHS 2017-2018**

“The ADHS surveys were done on a nationally representative sample that was representative at the prefecture level as well by rural and urban areas. A total of 715 enumeration areas (EAs) were selected as sample clusters, with probability proportional to each prefecture’s population size.The sample design called for 24 households to be randomly selected in every sampling cluster, regardless of its size, but some of the EAs contained fewer than 24 households. In these EAs, all households were included in the survey. The EAs are considered the sample’s primary sampling unit (PSU). The team of interviewers updated and listed the households in the selected EAs. Upon arriving in the selected clusters, interviewers spent the first day of fieldwork carrying out an exhaustive enumeration of households, recording the name of each head of household and the location of the dwelling. The listing was done with tablet PCs, using a digital listing application. When Interviewers completed their respective sections of the EA, they transferred their files into the supervisor's tablet PC, where the information was automatically compiled into a single file in which all households in the EA were entered. The software and field procedures were designed to ensure there were no duplications or omissions during the household listing process. The supervisor used the software in his tablet to randomly select 24 households for the survey from the complete list of households. All women aged 15-49 who were usual residents of the selected households or who slept in the households the night before the survey were eligible for individual interviews with the full Woman’s Questionnaire. Women aged 50-59 were also interviewed, but with an abbreviated questionnaire that left out all questions related to reproductive health and mother and child health. A 50% subsample was selected for the survey of men. Every man aged 15-59 who was a usual resident of or had slept in the household the night before the survey was eligible for an individual interview in these households.”

*Source: Institute of Statistics, Institute of Public Health, and ICF. 2018. Albania Demographic and Health Survey 2017-18. Tirana, Albania: Institute of Statistics, Institute of Public Health, and ICF. Available at: https://dhsprogram.com/pubs/pdf/FR348/FR348.pdf*

# **Algeria STEPS 2016**

“A multi-stage cluster sample of households. One individual within the age range of the survey was selected per household.”

*Source: NCD Microdata Repository. Study Description. Available at: https://extranet.who.int/ncdsmicrodata/index.php/catalog/91/study-description#page=sampling&tab=study-desc*

# **Azerbaijan STEPS 2017**

“A multi-stage cluster sample of households. One individual within the age range of the survey was selected per household.”

*Source: NCD Microdata Repository. Study Description. Available at: https://extranet.who.int/ncdsmicrodata/index.php/catalog/127/study-description#page=sampling&tab=study-desc*

# **Bangladesh STEPS 2018**

# “The sampling frame for the survey was the complete list of Primary Sampling Unit (PSU) i.e. Enumeration Areas (EAs) (about 293,533) covering the whole country prepared by the BBS for the 2011 Population and Housing Census of the People’s Republic of Bangladesh. A PSU is a geographic area covering 100 to 220 households with an average of 113 households. The sampling frame contained information about the PSU location, type of residence (urban or rural), and the estimated number of residential households. A sketch map that delineates the PSU geographic boundaries was available for each PSU. The population coverage rate of this Census 2011 was around 95.85% of the total population. (Annexure A)

# A special zonal operation was carried out by BBS before 2011 census in 2010 whereby both the urban and rural areas were subdivided with updating of mauzas (rural) and mahallas(urban) maps with demarcation of PSU boundaries comprising of 100 to 120 (average) houses. Thus based on 2011 census, the sampling frame for the survey was about 293,533 PSUs for both rural and urban areas. The urban stratum included urban and city corporation areas. In Bangladesh, 23.3% of the households are in urban areas; 8.2% are in city corporations, and 15.1% are in other than city corporations.

# A new division has been added in 2014 after conclusion of census 2011. So, all the PSUs in the 2011 census were mapped out as per the latest divisions. Thus the sampling frame for STEPS survey 2018 in Bangladesh comprised of 293,533 PSUs: 65,193 urban and 228,340 rural PSUs. […] Households in this survey was defined according to BBS as “A dwelling in which persons either related or unrelated living together and taking food from the same kitchen”.

# 2.3.3 Sampling strategy

# This survey used the same 496 PSUs which were sampled and used during a recently concluded GATS-II survey. In GATS Bangladesh 2017 these PSUs were equally allocated to each division (62 each), and within each division, were equally allocated to urban and rural stratum (248 PSUs each to both urban and rural strata). The rural and urban PSUs were arranged by population size in terms of household numbers for both urban and rural stratum in each division. In each stratum (rural and urban), 31 PSUs were selected independently in each division by probability proportional to size (PPS) sampling

# A household listing operation was carried out in all the selected PSUs by BBS during GATSII survey in July 2017 was used and no new household listing was carried out for this survey. As the survey used the same PSUs as used during GATS-II survey, HHs lists prepared by BBS during GATS-II survey in July 2017 served as sampling frame for the selection of households in the second stage.

# A fixed number of 20 households were systematically selected from each sampled PSU with an equal probability using a fractional interval technique. Selected households in all the selected PSUs were randomly assigned as “male” or “female” in a ratio that produced equal numbers of male and female households. The 20 selected HHs in a PSU were divided into two groups as 1) male HHs for interview of a male member and 2) female HHs for interview of a female member. All the sampled HHs from each PSU were listed sequentially, and alternate house was assigned as female or male household, with the first household in the list assigned as female household.

# Finally, one individual was sampled randomly from all the eligible adults in a participating household using the survey app in android tablets. No replacements and no changes of the pre-selected households were allowed at the implementing stage to prevent bias.”

# *Source: National Institute of Preventive and Social Medicine (NIPSOM) (2022) National STEPS Survey for Non-communicable Diseases Risk Factors in Bangladesh 2018*

# **Belarus STEPS 2016**

“At the first stage of sampling, the sampling units in each stratum (urban / rural area) were enumeration plots formed for organizing the 2009 census. Within each region, strata (urban / rural area) from the total list of enumeration plots using systematic sample procedures with probability proportional to the size of the 2009 census plots, enumeration plots were selected. PSUs were selected independently for each stratum (urban / rural) within each region and city of Minsk using the following sample procedures: All census enumeration areas for the 2009 census of the Republic of Belarus were grouped within each region by urban and rural areas. Thus, a total of 13 counting plots were formed (2 groups in 6 regions and 1 group in Minsk). We will call each of these sets of enumeration plots a sampling segment. The enumeration plots within each sampling segment were ranked in the order of their geographical location, which was determined as a result of drawing a serpentine line on a geographical map. In order to cover the entire territory of the republic, a curve was drawn on the map, which in a serpentine order divided the territory of each region into layers, which were then assigned serial numbers depending on their geographical location. In all regions (except the city of Minsk), counting sections were assigned the number of the layer in which the corresponding settlement is located. In the city of Minsk, implicit stratification took place by means of a serpentine distribution directly of enumeration plots throughout the city, taking into account its administrative-territorial division. The ranking of the enumeration plots was carried out by increasing the number of the layer. For the size of the enumeration plot, information is drawn from to the 2009 census regarding the population in it (the number of members of private households, with the exception of HH located in closed areas). Using the ordered list of enumeration sites, the accumulated (cumulative) values ​​of the enumeration area size (indicator “population”) were calculated. The final cumulative value represents the total population in the sample segment. (...) Using the "Random Number" function, a random value from 0 to 1 (Rh) is selected. The first selected enumeration area was determined by multiplying the selection step (Sh) by a random value (Rh) and comparing this value with the cumulative value of the “population” (Qh) indicator. Based on the selection step, all subsequent selected PSUs were determined. The selected counting sections i in the sample segment h were determined by comparing their cumulative values ​​(Qhi) and the value (Uhi) (...)

The first stage of selection was completed by selecting the required number of PSUs in each of the seven regions, separately for urban and rural areas. In the republic as a whole, with different probabilities, 288 counting plots were selected: 144 in urban and 144 urban areas. (...) The selection of HHs was carried out within each selected counting area (PSU). The basis for the selection is a list of private households that include persons aged 18-69 years, indicating the addresses of residential apartments and households. (...) After that, from a list of HHs in each selected PSU, a fixed number of HHs was systematically selected, equal to 20, which corresponds to the established cluster size. The start of selection was determined randomly (using the "Random Number" function). Given the fact that the sizes of the counting sections are close, but all are different since the number of selected units is fixed, then in each PSU a new interval and a new random start of selection were calculated. In general, 5760 HHs were selected in the republic.”

*Source, translated from: Prevalence of Risk Factors of Non-Infectious Diseases in the Republic of Belarus STEPS 2016. Available at: https://www.who.int/ncds/surveillance/steps/belarus/en/*

**Belize CAMDI 2010**

“The study utilized a cross-sectional, household survey design, with multistage stratified random cluster sampling…The study used a national representative sample of the adult population (20 years of age and older) in the six districts of the country. Districts are divided into smaller units called enumeration districts (EDs). The primary sample unit for survey was the ED. District sample sizes were determined proportionate to the size of the district population in relation to the national population (Table B). From each district a random 10% of EDs were selected from which to select households (Table B). Within each ED, household clusters were randomly selected utilizing a grid developed by the Central Statistical Office. A household was defined as one or more persons living together i.e. sleeping at least four nights per week AND sharing at least one daily meal with the household.”

*Source: Ethan Gough, Englebert Emmanuel, Valerie Jenkins, Lorraine Thompson, Enrique Perez, Alberto Barcelo. The Central American Diabetes Initiative: Survey of Diabetes, Hypertension, and 17 Non-communicable Disease Risk Factors. Belize. 2008. Available from:* [*http://www1.paho.org/hq/dmdocuments/2010/CAMDI_Report_BELIZE_0810.pdf*](http://www1.paho.org/hq/dmdocuments/2010/CAMDI_Report_BELIZE_0810.pdf)

# **Benin STEPS 2015**

“The study was conducted using a three-stage random sampling technique. The sampling frame was provided by the National Institute of Statistics and Economic Analysis (INSAE) from the data of the fourth General Census of Population and Housing (RGPH4) in Benin in 2013. The first stage consisted of the random selection of 260 Enumeration Areas (EAs);  The second stage consisted in drawing lots for 20 households per EA;  The third step consisted in randomly selecting one individual per household retained according to the Kish method recommended by the WHO for the STEPS survey.”

*Source, translated from: Rapport final de l’enquête pour la surveillance des facteurs de risque des maladies non transmissibles par l'approche ‘’STEPSwise’’ de l'OMSENQUETE ‘’STEPS 2015’’ au Bénin. Available at: https://www.who.int/ncds/surveillance/steps/benin/en/.*

# **Bhutan STEPS 2019:**

“To achieve a nationally representative sample, a multi-stage sampling method was used to select enumeration areas, households and eligible participants at each of the selected households in three stages.

Sampling frame: For this STEPS survey the sampling frame was used from the 2017 Population and Housing Census of Bhutan (PHCB).

Stage 1: Type and Number of PSUs selected:

The Primary Sampling Units (PSUs) of this survey was Gewogs in rural areas and towns in urban areas. Overall 88 PSUs were selected which comprised 33 from urban and 55 from rural areas. PSUs from each stratum (urban and rural) of each region were selected using Probability Proportionate to Size (PPS).

Stage 2: Selection of Secondary Sampling Units (SSUs)

The Secondary Sampling Units of this survey was chiwogs in rural areas and enumeration areas (EAs) in urban areas. Four SSUs were selected for every PSU which led to the selection of 352 SSUs (220 from rural and 132 from the urban). This was also selected using PPS sampling by using the number of households in each SSU.

Stage 3: Selection of households from SSU

Within each SSU, 16 households were selected using circular systematic sampling. The sampling frame for this was the list of households with a unique identification number (ID) developed by the enumerators during the survey. The household listing was done by the enumerators under the supervision of the team leader/supervisor with the help of Chiwog Tshogpa or Thromde Thuemi or local healthcare providers.

Stage 4: Selection of eligible participants at the household level

At the household level, Kish sampling method was used to randomly select one eligible member aged 15-69 years for the survey. The Kish method ranks eligible household members in order of decreasing age, starting with males and then females, and randomly selected a respondent using an automated program for Kish section in the handheld android Samsung tablets.”

*Source: Department of Public Health, Ministry of Health, (2020). Non-communicable disease Risk Factors: Bhutan STEPS Survey 2019, Thimphu*

# **Botswana STEPS 2014**

“Botswana has a population of over 2 million with 27 districts and 4,845 enumeration areas and sample size of 300 enumeration areas with a target population of 6,400 people was systematically drawn from a pool of the whole enumeration areas.  Against the identified enumeration areas numbers of households were listed and proportion of participants was calculated from the total sample size required for the country.  Finally a computer generated random number was drawn to go into specific households in that specific enumeration area and at the end eligible participants residing in the household were listed into the electronic hand held data assistant(PDA) and at the end a name was picked automatically to participate in the survey.”

*Source: Botswana STEPS Survey Report on Non-communicable Disease Risk Factors - December 2015. Available at: https://www.who.int/ncds/surveillance/steps/botswana/en/.*

**Brazil Pesquisa Nacional de Saúde 2013**

“The Master Sample is a set of units of areas that are selected to meet various surveys of the IBGE Integrated System of Household Searches (SIPD). These units are considered primary sampling units (PSUs) in the sample planning of each of the surveys that use the Master Sample, such as PNS. The sampling plan consists of the stratification of the UPAs and selection of these units with probability proportional to the size, given by the number of permanent private households (DPPs).

The register for selection of the Master Sample was a file containing information from the Demographic Census 2010 on the census tracts of the geographic scope, whose limits are defined in the Operational Geographic Base 2010, totaling 316574 sectors. A sector or set of sectors with at least 60 DPPs was defined as UPA, with the exception of a few units, because it was not possible to aggregate sectors in some municipalities.

The stratification of the UPAs obeys four different criteria: administrative, including the division of the UF into capital, rest of the Metropolitan Region (RM) or Integrated Region of Economic Development - RIDE, and rest of the UF; geographical subdivision, which subdivides capitals and other large municipalities into more strata; situation that involves rural / urban categorization; and the statistician in order to improve the accuracy of the estimates.

As part of the SIPD, the sampling design of the PNS followed, in part, the sampling design of the Master Sample, especially with regard to the stratification of the UPAs.

The PNS sample is by clusters in three stages of selection:

1st stage: selection with probability proportional to the size (given by the number of DPPs in each unit) of the UPAs sub-sample in each stratum of the Master Sample;

2nd stage: selection by simple random sample of households in each UPA selected in the first stage;

3rd stage: selection by simple random sampling of the adult (person aged 18 years or older) among all adult residents of the household.

**Burkina Faso STEPS 2013**

“Sampling methodology: The study was conducted on a sample obtained from a three-stage cluster stratified as recommended by the WHO for STEPS screening surveys. risk factors for noncommunicable diseases. The sampling frame used was that derived from the general census of the population and habitat 2006 (RGPH 2006) and updated in 2010 during the survey Demographic and Health Survey of Burkina Faso (EDS-BF, 2010). This update concerned the enumeration areas (EAs) that correspond to the cluster as part of this study.

Selection of clusters: The choice of clusters was made according to a systematic random selection proportional to their size (in number of households) within strata (regions). To do this clusters were organized by stratum and place of residence (urban / rural). A total of 240 clusters of which 185 were in rural areas and 55 in urban areas were selected for the investigation. Selection of households: Households were randomly drawn after an enumeration exhaustive list of all households in the cluster. A draw tool designed on Excel by the team. The technique was used in the field for selecting households to investigate. In total, 20 households in clusters were selected to participate in the study.

Selection of individuals: The choice of individuals was made randomly using Kish's method. In total, an individual aged 25 to 64 living in a selected household was fired for participate in the survey.”

# *Source, translated from: Rapport de l’enquete national sur la prevalence des principaux facteurs de risques communs aux maladies non transmissibles au Burkina Faso Enquete STEPS 2013. Available at:* [*http://www.who.int/chp/steps/burkina_faso/en/*](http://www.who.int/chp/steps/burkina_faso/en/)*.*

# **Cabo Verde STEPS: 2011**

“The sample was selected following a three-stage probabilistic sampling, namely:

a) the Census District (DR) as the primary sampling unit,

b) the household as a secondary unit,

c) the individual as a tertiary unit.

The DR was selected through the random method, proportional to the size of the accommodations, and in each selected DR a fixed number of 20 households were selected through the systematic method. The third unit was selected automatically, through the Kish method, within each selected household.“

*Source: Translated from: Ministério da Saúde, Instituto Nacional de Estatística, Organização Mundial da Saúde Cabo Verde, Segundo Inquérito Nacional sobre os Fatores de Risco das Doenças Não Transmissíveis (IDNT II)*

# **Cambodia STEPS 2010**

“The survey was designed to cover all geographical areas of Cambodia and a 3-stage sampling process as part of the multi-stage cluster sampling was carried out to randomly select the target population: random selection of communes (Khum in rural areas and its equivalent Sangkat in urban area) as primary sampling unit (PSU), followed by villages (Phum) for the secondary sampling unit (SSU), and by households for the elementary unit (EU). Finally all members of the randomly chosen households aged 25-64 years were invited to participate in this survey. The selection process was performed identically for urban and rural areas in order to get a self-weighted estimate for the whole population of the country. A total of 180 clusters with 34 clusters from the urban area and 146 clusters from the rural area were randomly selected.”

*Source: Prevalence of Non-Communicable Disease Risk Factors in Cambodia - STEPS survey Country Report, September 2010. Available at: https://www.who.int/ncds/surveillance/steps/cambodia/en/.*

**China Health and Nutrition Survey 2009**

“The China Health and Nutrition Survey is a longitudinal study across 228 communities within nine provinces of China. Surveys began in 1989, with subsequent surveys every 2–4 years, for a total of nine rounds between 1989 and 2011. The China Health and Nutrition Survey was designed to provide representation of rural, urban and suburban areas varying substantially in geography, economic development, public resources and health indicators,13 and it is the only large-scale, longitudinal study of its kind in China. The original survey in 1989 used a multistage, random cluster design in eight provinces (Liaoning, Jiangsu, Shandong, Henan, Hubei, Hunan, Guangxi and Guizhou) to select a stratified probability sample; a ninth province, Heilongjiang, was added in 1997 using a similar sampling strategy. Essentially, two cities (one large and one small city— usually the provincial capital and a lower income city) and four counties (stratified by income: one high, one low and two middle income counties) were selected in each province. Within cities, two urban and two suburban communities were selected; within counties, one community in the capital city and three rural villages were chosen. Twenty households per community were then selected for participation. The study met the standards for the ethical treatment of participants and was approved by the Institutional Review Boards of the University of North Carolina at Chapel Hill and the Institute of Nutrition and Food Safety, Chinese Center for Disease Control and Prevention.”

*Source: Attard, Samantha M.; Herring, Amy H.; Wang, Huiling; Howard, Annie Green; Thompson, Amanda L.; Adair, Linda S.; Mayer-Davis, Elizabeth J.; & Gordon-Larsen, Penny. (2015). Implications of Iron Deficiency/Anemia on the Classification of Diabetes Using HbA1c. Nutrition & Diabetes, 5, e166.*

**Chile National Health Survey 2009-2010**

“The sampling frame was constituted from the Population and Housing Census 2002. The design of the study was transversal, with a random sample of complex type households (stratified and multi-stage by clusters) with national, regional and area representation rural / urban. The target population was adults older than or equal to 15 years. The survey had a response rate in the eligible population of 85%. The refusal rate was of 12%. 5,434 people were interviewed. A nurse performed clinical and examinations to 5,043 participants and 4,956 accepted laboratory tests (blood and urine). The total sample loss of the oversized sample was 28% (this including rejection, non-contact and other causes of random loss). The raw sample was designed with overrepresentation of some population groups (older adults, regions other than the Metropolitan Region and rural areas) to increase sample efficiency and homogenize the accuracy of the estimators. The expansion of the sample data is because it grants each participant the weight that corresponds to it according to the design sample and at the same time corrects the distortion of the raw sample, making it coincide with the census population projection for January 2010 for Chilean adults over 15 years of age.”

*Source, translated from: Resumen Ejecutivo: Encuesta Nacional de Salud ENS Chile 2009-10. Available at:* [*http://epi.minsal.cl/encuesta-ens-anteriores/*](http://epi.minsal.cl/encuesta-ens-anteriores/)

**Comoros STEPS 2011**

“The STEPS survey on risk factors for chronic diseases in the Union of the Comoros took place from January to March 2011. This study has undertaken Step 1, Step 2 and Step 3. Indeed, sociodemographic and behavioral measures were collected in Step 1. Physical measures such as height, weight and tension were collected in Step 2 and biochemical measurements were collected to assess the levels of blood glucose and cholesterol levels in Step 3. The STEPS survey conducted in Comoros Union is a survey of general population, targeting adults aged 25 to 64 years. A stratified survey was used to produce representative data for this age group. A total of 5556 adults aged 25 to 64 participated in the STEPS survey on a sample of 5760 people representing an overall response rate of 96.5%.”

*Source, translated from : Union des Comores. STEPS 2011 - Note de synthèse. Available at:* [*http://www.who.int/chp/steps/comoros/en/*](http://www.who.int/chp/steps/comoros/en/)*.*

**Costa Rica STEPS 2010**

“The Costa Rican NCRFSS survey was a cross-sectional survey based on a probabilistic cluster sampling design. The NCRFSS survey was conducted during 2010 under the supervision of the Caja Costarricense de Seguro Social, a government public healthcare provider, and covers the overall adult population aged ≥20 years. Multistage cluster sampling was performed stratified by geographical areas, age groups (20–39, 40–64, and ≥65 years) and gender. The first sample stage was the randomized selection of the country’s geographical areas as primary sample units followed by the random selection of sectors in selected areas as secondary sample units. The random selection of areas and sectors was performed with probability proportional to size; the area or sector size was determined by the population >20 years during 2009, as estimated by the Costa Rican Census and Statistics National Institute (INEC). Households were chosen through a random number generator using dwelling lists obtained from the health technician assistant in every community until all age group and gender strata sample sizes were achieved. A family dwelling was defined as a group of people who share the same table to eat. Survey participants were selected by the Kish method, which samples participants within a household with equal probability of selection, as recommended by the WHO STEPwise methodology. To be eligible for inclusion in the study, subjects had to be ≥20 years of age, permanently residing in the selected homes, and to have provided written consent. Pregnant or lactating mothers and those who were within 6 months postpartum were excluded from the study. Each participant selected for the study was informed of the study objectives and details before agreeing to participate in the investigation. In all, 3653 noninstitutionalized adults were surveyed, with an 87.8% response rate of the eligible population.”

# *Source: Wong-McClure R, Gregg EW, Barcelo A, Sanabria-Lopez L, Lee K, Abarca-Gomez L, Cervantes-Loaiza M, Luman ET. Prevalence of diabetes and impaired fasting glucose in Costa Rica: Costa Rican National Cardiovascular Risk Factors Survey, 2010. J Diabetes. 2016 Sep;8(5):686-92.*

# **Costa Rica STEPS 2018:**

“The survey was conducted between June and December 2018 and was applied by the Technical Assistants in Primary Care (ATAP) of the selected localities. The report presents the results of the third nationwide determination of the prevalence of cardiovascular disease risk factors, which was preceded by the baseline determination in 2010 and, subsequently, by the second determination in 2014. For all determinations, the methodology used for this survey was based on the STEPS method, proposed by the World Health Organization (WHO) and the Pan American Health Organization (PAHO), which contemplates the evaluation of behavioral risk factors such as physical activity and the consumption of fruits, alcohol, salt and tobacco; in addition to biological risk factors such as diabetes, arterial hypertension and dyslipidemia at the population level. The use of the same methodology for the current survey in relation to previous surveys makes it possible to establish valid comparisons between them.”

*Source: Translated from: Dirección Desarrollo de Servicios de Salud. Vigilancia de los factores de riesgo cardiovascular, tercera encuesta 2018.*

# **Egypt DHS 2015:**

“The 2015 EHIS took advantage of the sample developed for the ever-married women survey component of the 2014 EDHS. The 2014 EDHS was implemented in a total of 842 primary sampling units (PSUs) selected from 25 governorates. The frame for selection of these units was a list of all shiakhas and villages in Egypt. This list was obtained from the Central Agency for Public Mobilization and Statistics (CAPMAS) and updated as necessary to reflect any recent changes.

For the EHIS, a sub-sample of 614 PSUs (shiakhas/villages) was selected from the 842 PSUs included in the 2014 EDHS sample. The household listing prepared during the 2014 EDHS for these PSUs was used to select the household sample for the 2015 EHIS. The selection was conducted in such a way that the EHIS household sample was totally independent of the 2014 EDHS sample, i.e., no household was included in both samples. It was expected that approximately 28,500 individuals age 6 months to 59 years, eligible for the 2015 EHIS testing and interviews, would be identified in the selected households.

The sample for the 2015 EHIS was designed to provide estimates of the key health indicators that the survey was designed to measure including the prevalence of hepatitis B and C for the country as a whole and for six major subdivisions (Urban Governorates, urban Lower Egypt, rural Lower Egypt, urban Upper Egypt, rural Upper Egypt, and Frontier Governorates). The sample also allows for estimates of some key indicators at the governorate level. Additional information on the sample design and implementation is provided in Appendix A.”

*Source: Ministry of Health and Population [Egypt], El-Zanaty and Associates [Egypt], and ICF International. 2015. Egypt Health Issues Survey 2015. Cairo, Egypt and Rockville, Maryland, USA: Ministry of Health and Population and ICF International.*

# **El Salvador ENECA 2015**

“A two-staged cluster sample design was employed with stratification of first-stage units. Primary sampling units (PSU) almost always corresponded to census tracts, though sometimes several tracts were joined to yield PSUs of similar size. Each sample segment included 150–250 households. Stratification prior to PSU selection was based on urban/rural residency, as well as geographic distribution throughout the country’s five regions according to the latest national population and households census conducted in 2007. In selected PSUs within each of the ten strata formed by the crossing of these two axes, households were selected (secondary sampling units) and, finally, all individuals corresponding to the target population residing in them were included in the sample. In each of its phases, selection was carried out applying probabilistic techniques

*Source: Orantes-Navarro, Carlos Manuel, Miguel M. Almaguer-López, Patricia Alonso-Galbán, Moisés Díaz-Amaya, Samuel Hernández, Raúl Herrera-Valdés, and Luis Carlos Silva-Aycaguer. "The chronic kidney disease epidemic in El Salvador: a cross-sectional study." MEDICC review 21 (2019): 29-37.*

# **Ethiopia STEPS 2015**

“A single population-proportion formula was implemented to determine the sample size. To adjust for the design effect, a complex sampling design effect coefficient of 1.5 was used to compute the sample size. In order to have an adequate level of precision for each age-sex estimate and place of residence, the sample was multiplied by the number of age-sex and place of residence groups for which the estimates were reported. Thus, Z-score=1.96; proportion =35.2% (11); marginal error=0.04; design effect =1.5; age-sex estimate and place of residence - sex estimate =10 groups, and non-response rate=20%. Thus, 10,260 study participants were to be included in the study. In this study, a mix of sampling approach namely stratified, three-stage cluster sampling, simple random sampling and Kish method were employed to select the study settings and the study participants. The sampling frame was based on the population and housing census conducted for Ethiopia in 2007 (CSA, 2008). There are 11 regions including the two city administrations (Addis Ababa and Dire Dawa). Each region is divided into administrative zones. The two city administrations are divided into sub-cities. The administrative zones in the nine regions and sub-cities in the two city administrations are subdivided into districts or ‘Woreda’. The districts ‘Woredas’ are also further divided into ‘Kebele’. The kebeles are the smallest administrative units with clear geographic jurisdiction in Ethiopia. Within Kebeles, there are Enumeration Areas (EAs) which are delineated by the Central Statistical Agency (CSA) of Ethiopia. Therefore, Enumeration Areas (EAs) were considered as the primary sampling units for this survey. According to the 2007 population and housing census, there were a total of 15,837 Kebeles in Ethiopia i.e. 14,364 in rural and 1,473 in urban kebeles(12). Taking into account the cost of the study and the level of precision, 20 households per EA and one eligible individual from each household with a total of 513 EAs were covered nationwide. Stratifying the sampling design by place of residence we allocated about 404 EAs for rural and the remaining 109 to urban areas (Table 2). As shown in the table 2.1 above, the Primary Sampling Units (PSUs) were the EAs both in rural and urban settings. At the first stage, 513 PSUs (404 rural and 109 urban) were selected with probability proportionate to size. This is followed by a random selection of secondary sampling units (SSUs) per selected PSU in the second stage. The Secondary Sampling Units (SSUs) were the households. The total number of EA in 2007 was 82,037. The Enumeration areas essentially comprise on average 100 households both in urban and rural settings. Twenty households were selected from each EA using systematic sampling. Thus, a total of 10,260 households were selected from the 513 EAs (20 households per EA). The sampling interval was determined by dividing the total number of households in the selected EA by 20. Prior to sampling, supervisors and data collectors visited the selected EAs and conducted a fresh listing of all households in that EA in consultation with local health workers and any other active member who have a good understanding of the local context. In the third stage, eligible individuals were selected from household using Kish method. Only one eligible participant (an adult age 15-69 years) in the selected household was enrolled in the survey. Using the Kish method, eligible participants in each household were ranked in order of decreasing age, starting with men followed by women.”

*Source: Department of Public Health, Ministry of Health (2020). Non-communicable disease Risk Factors: Ethiopia STEPS Survey 2015*

# **Eritrea STEPS 2010**

“A multi-stage cluster sample of households. One individual within the age range of the survey was selected per household.”

*Source: NCD Microdata Repository. Study Description. Available at: https://extranet.who.int/ncdsmicrodata/index.php/catalog/589/study-description#page=sampling&tab=study-desc*

# **Fiji STEPS 2011**

“A multi-stage cluster sampling methodology was used with primary sampling units being the enumeration areas (EA) or clusters. The secondary units were the households from which eligible participants were selected (three stages).

First Stage: Fiji’s entire population is divided administratively into 4 main divisions and further subdivided into 14 provinces plus the self-governing island of Rotuma. All main divisions were included in the survey excluding Rotuma due to its remote location. Each province is divided into tikinas (administrative units) and further subdivided into enumeration areas (EA) by the Fiji Bureau of Statistics (FBOS). Fiji has a total of 86 tikinas and 1602 EAs. After excluding Rotuma, 42 tikinas were selected proportional to the size of each province’s population. This represented >95% of Fiji’s population. The first stage of the sampling process involved the selection of EAs proportional to the size of each Tikina.

Second Stage: The second stage of the sampling process involved the selection of 50 households from the selected EAs through simple random selection.

Third Stage: Within each household, the selection of one eligible participant (aged 25 -64 years) for STEPS 1 and 2 was performed using the KISH method. Random selection was conducted after all eligible members (males and females) in each household were listed in descending order of age starting with males and then females.

For each EA, data was also collected regarding the total number of households approached, the number of those who refused to participate, and the number of those in STEP 1 who also participated in STEP 2 and STEP 3. This data was used to calculate sampling weights for data analysis.”

*Source: Ministry of Health and Medical Services, (2018). Fiji NCD Risk Factors: STEPS REPORT 2011, Suva.*

# **Gambia STEPS 2010**

“The STEPS survey in The Gambia was a population-based survey of adults aged 25-64. Probability proportional to size sampling was used in both the first and second stage of sampling to produce representative data for that age range in The Gambia. A total of 5,280 people were targeted for the study but only a total of 4,111 participated.  The overall response rate was thus 77.9%.”

*Source: The Gambia STEPS Survey 2010 Fact Sheet. Available at: https://www.who.int/ncds/surveillance/steps/gambia/en/*

**Georgia STEPS 2016**

“The STEPS survey of non-communicable disease (NCD) risk factors in Georgia was carried out from June 2016 to September 2016. Georgia carried out Step 1, Step 2 and Step 3. Socio demographic and behavioural information was collected in Step 1. Physical measurements such as height, weight and blood pressure were collected in Step 2. Biochemical measurements were collected to assess blood glucose and cholesterol levels in Step 3. The survey was a populationbased survey of adults aged 18-69. A Multi-stage cluster sampling design was used to produce representative data for that age range in Georgia. A total of 5554 adults participated in the survey. The overall response rate was 75.7%. The following equipment was used for the biological measurements: Samsung Galaxy Tab 4OS; Cardiochek PA; Growth Management Scale (330 HRS BMI); BMI calculator Seca 491; ergonomic Seca 201; test panels (Chol/HDL/Glu for Cardiocheck PA); and the blood pressure monitor Bosch Sohn Medicus UNO.”

Source: Georgia STEPS Survey 2016 Fact Sheet. Available at: <http://www.who.int/chp/steps/georgia/en/>.

**Ghana SAGE 2007-8**

“The sampling method used for the Ghana SAGE Wave 1 was based on the design for the World Health Survey, 2003, in which the primary sampling units (PSUs) were stratified by region and location (urban/rural). Selection of the PSUs was based on proportional allocation by size. Each enumeration area (EA) was selected independently within each stratum. In the WHS/SAGE Wave 0, a total of 6 000 households were to be interviewed and therefore 300 EAs were selected nationwide. Twenty households were to be randomly selected in each EA using systematic sampling. The number of EAs per region was based on the population size of the region. For SAGE Wave 1, a total of 5 000 50+ respondents and 1 000 18–49-year-old respondents were required and therefore 250 EAs out of the 298 EAs of the WHS/ SAGE Wave 0 were used based on the availability of respondents aged 50+ years within the EAs.

Enumeration areas with no 50+ individuals were not included. Within each EA, 20 households with one or more 50+ individuals and four households with members aged 18–49 were to be selected. All respondents aged 50+ within households with over 50s from the WHS were automatically selected and additional households with members aged 50+ years were randomly selected to make a total of 20 households for each EA. The four households of the 18–49 years age group were randomly selected from the WHS/SAGE Wave 0 households list per EA. All the 50+ year olds within the selected households were to be interviewed together with the four identified under-50 respondents. Field work and data entry were undertaken between May 2007 and June 2008.”

*Source: Richard Biritwum, George Mensah, Alfred Yawson and Nadia Minicuci. Study on global AGEing and adult health (SAGE) -Wave 1: The Ghana National Report. University of Ghana Medical School, Department of Community Health. July 2013*

**Grenada STEPS 2010-11**

“The sample frame comprised adults 25 to 64 years throughout Grenada, Carriacou and Petite Martinique. Administratively, the state of Grenada is divided into seven parishes with the islands of Carriacou and Petite Martinique being one parish. Each parish has a town (with the exception of St. David’s) and several villages.A three-stage stratified sampling methodology was constructed using the Population and Housing Census 2001 as the sampling frame. The master frame was divided into 42 regions with an average size of eight (800) hundred households per region using a contiguous set of Enumeration Districts (EDs), where the approximate size of each ED is between 46 – 189 households (refer to Table 36 in the appendix). In the first stage, a paired design, i.e. samples of two EDs were randomly selected per region using a 3-digit table of random numbers. Since the frame was stratified by parish it would mean that in the large parishes, a larger number of EDs were selected. Since the ED size was fairly consistent, there was no need to use PPS sampling and hence simple random sampling was used. The number of households enumerated per ED was calculated ((1736/42)/2) =21 There were therefore twenty-one households per selected ED. At the second stage, the sampling interval was computed by dividing the total number of households in the selected ED by the determined sample size of twenty-one (21) households per ED. Once the ED had been listed and the listing returned to the CSO a random table was used to select a random number (k) between 1 and the sample interval value, I, inclusive then to this number was added the sampling interval for the full list of households within the ED. Thus, the list of selected households was k, k+I, k+2I, … k+(n-1)I where n is the size assigned to each ED (21). The third stage of the sampling required a listing of the members of the selected household then using the KISH Method the eligible person to be interviewed was selected.”

*Source: Grenada STEPS team. Grenada WHO STEPS Country Report 2010-2011. Available at:* [*http://www.who.int/ncds/surveillance/steps/grenada/en/*](http://www.who.int/ncds/surveillance/steps/grenada/en/)

**Guyana STEPS 2016**

“A response rate of 66.68% will be selected based on the experience and response rates of other surveys over the years such as the recent Demographic Health Survey 2009. [...] STEPS 3 involve taking blood samples from a proportion of the sample, in this case 50% of the sample, in order to measure raised blood glucose levels and abnormal blood lipids. [...] The STEPS sample will be prepared by the Bureau of Statistics Guyana following the recommended STEPS sample methodology. A multi-stage cluster sampling design will be used. Guyana is divided into 10 administrative regions and within the administrative regions there are seven towns and each region is further divided into enumeration districts. For the STEPS survey 288 enumeration districts will be selected using the population probability sampling method and from each enumeration district 12 households will be selected giving a total sample size of 3456. Further at the household level each participant will be randomly selected by the electronic tablet. For STEP 3 50% of the sample will be randomly selected to participate. A re-listing of some households may also be necessary, such as those interior region locations, in which case in addition to household listings, enumeration districts maps will also be provided so that a re-listing can be done where required.”

*Source: STEPwise Approach to Chronic Disease risk factor surveillance (STEPS): Guyana’s Implementation Plan. June 20, 2016. Ministry of Public Health, Guyana.*

# **Haiti DHS 2016-2017**

“A national sample of 13,546 households was selected, of which 13,405 were interviewed. The sample is stratified so as to provide an adequate representation of the urban and rural areas as well as of the 11 fields of study, corresponding to the 10 departments, and to the Metropolitan Area. The EMMUS-VI (Sixth Mortality, Morbidity and Service Use Survey) sample is a stratified area sample, drawn in two stages. In the first stage, 450 Enumeration Sections (SDE) or clusters were drawn across the national territory, with a systematic drawing with probability proportional to the size. The size of an SDE is the number of households residing in the SDE. The sampling frame used for EMMUS-VI is the 2003 General Population and Housing Census (RGPH 2003), with a partial update in 2011 by the IHSI. After the EDS was drawn and before the main survey, a household count and an update of the map of each selected EDS were carried out. In the second stage, a sample of households was drawn with a systematic drawing with equal probability from the newly established list in the enumeration. All women aged 15-49 usually living in selected households, or present the night before the survey, were eligible to be surveyed. In two thirds of households, the survey was also carried out among men aged 15-64. In a third of households, women aged 50-64 and men aged 35-64 were also eligible, but only for certain aspects of the survey.”

*Source, translated from: 2018. Enquête Mortalité, Morbidité et Utilisation des Services - EMMUS-VI 2016-2017 Pétion-Ville, Haïti, et Rockville, Maryland, USA : IHE et ICF. Available at: https://dhsprogram.com/pubs/pdf/FR326/FR326.pdf*

# **India NFHS 2019-2021**

Decisions about the overall sample size required for NFHS-5 were guided by several considerations, paramount among which was the need to produce indicators at the district and/or state/union territory (UT) levels. Thus, NFHS-5 provides information for 707 districts, 28 states, and 8 union territories.

A uniform sample design, which is representative at the national, state/union territory, and district level, was adopted in each round of the survey. Each district is stratified into urban and rural areas. Each rural stratum is sub-stratified into smaller substrata which are created considering the village population and the percentage of the population belonging to scheduled castes and scheduled tribes (SC/ST). Within each explicit rural sampling stratum, a sample of villages was selected as Primary Sampling Units (PSUs); before the PSU selection, PSUs were sorted according to the literacy rate of women age 6+ years. Within each urban sampling stratum, a sample of Census Enumeration Blocks (CEBs) was selected as PSUs. Before the PSU selection, PSUs were sorted according to the percentage of SC/ST population. In the second stage of selection, a fixed number of 22 households per cluster was selected with an equal probability systematic selection from a newly created list of households in the selected PSUs. The list of households was created as a result of the mapping and household listing operation conducted in each selected PSU before the household selection in the second stage. In all, 30,456 Primary Sampling Units (PSUs) were selected across the country in NFHS-5 drawn from 707 districts as on March 31st 2017, of which fieldwork was completed in 30,198 PSUs.

The NFHS-5 sample is a stratified two-stage sample. The 2011 census served as the sampling frame for the selection of PSUs. PSUs were villages in rural areas and Census Enumeration Blocks (CEBs) in urban areas. PSUs with fewer than 40 households were linked to the nearest PSU. Within each rural stratum, villages were selected from the sampling frame with probability proportional to size (PPS). In each stratum, six approximately equal substrata were created by crossing three substrata, each created based on the estimated number of households in each village, with two substrata, each created based on the percentage of the population belonging to scheduled castes and scheduled tribes (SCs/STs). Within each explicit sampling stratum, PSUs were sorted according to the prevalence of literacy of women age 6+ years. The final sample PSUs were selected with PPS systematic sampling.“

*Source: International Institute for Population Sciences (IIPS) and ICF. 2021. National Family Health Survey (NFHS-5), 2019-21: India. Mumbai: IIPS.*

**Indonesia Indonesia Family Life Survey 2014-15**

“Because it is a longitudinal survey, IFLS5 drew its sample from IFLS1, IFLS2, IFLS2+, IFLS3 and IFLS4. The IFLS1 sampling scheme stratified on provinces and urban/rural location, then randomly sampled within these strata (see Frankenberg and Karoly, 1995, for a detailed description). Provinces were selected to maximize representation of the population, capture the cultural and socioeconomic diversity of Indonesia, and be costeffective to survey given the size and terrain of the country. For mainly cost-effectiveness reasons, 14 of the then existing 27 provinces were excluded.3 The resulting sample included 13 of Indonesia’s 27 provinces containing 83% of the population: four provinces on Sumatra (North Sumatra, West Sumatra, South Sumatra, and Lampung), all five of the Javanese provinces (DKI Jakarta, West Java, Central Java, DI Yogyakarta, and East Java), and four provinces covering the remaining major island groups (Bali, West Nusa Tenggara, South Kalimantan, and South Sulawesi).

Within each of the 13 provinces, enumeration areas (EAs) were randomly chosen from a nationally representative sample frame used in the 1993 SUSENAS, a socioeconomic survey of about 60,000 households. The IFLS randomly selected 321 enumeration areas in the 13 provinces, oversampling urban EAs and EAs in smaller provinces to facilitate urban-rural and Javanese–nonJavanese comparisons.

Within a selected EA, households were randomly selected based upon 1993 SUSENAS listings obtained from regional BPS office. A household was defined as a group of people whose members reside in the same dwelling and share food from the same cooking pot (the standard BPS definition). Twenty households were selected from each urban EA, and 30 households were selected from each rural EA. This strategy minimized expensive travel between rural EAs while 43 balancing the costs of correlations among households. For IFLS1 a total of 7,730 households were sampled to obtain a final sample size goal of 7,000 completed households. This strategy was based on BPS experience of about 90% completion rates. In fact, IFLS1 exceeded that target and interviews were conducted with 7,224 households in late 1993 and early 1994.

In IFLS1 it was determined to be too costly to interview all household members, so a sampling scheme was used to randomly select several members within a household to provide detailed individual information.”

*Source: Strauss, J., F. Witoelar, and B. Sikoki. “The Fifth Wave of the Indonesia Family Life Survey (IFLS5): Overview and Field Report”. March 2016. WR-1143/1-NIA/NICHD.*

# **Iran STEPS 2016**

“For proportional to size sampling, we designed a systematic cluster random sampling frame through which 31,050 participants (3105 clusters) were selected from urban and rural areas of 31 provinces of Iran. To estimate the minimum sample at the 95% country with 384 samples (Ilam) was considered as the basis of calculations. The sample size of other provinces was calculated according to the population ratio of each to the referenced province. To consider the effect of sampling design and to control non–response error, 10% was added to the estimated samples of each province. With a view to reducing costs and increasing productivity, it was decided that for provinces with 800 or more samples through weighting methods, half of calculated sample size taken along with the twice weight in estimating. In this regard, national individual ID and postal code were used as part of individual characteristics in the questionnaire that has to be validated by interviewer through seeing national ID card.

The eligible population for study was defined according to the criteria of being among 18 years old Iranian adults that resided in Iran at the time of data collection. The first and second steps of study have been run for all selected samples and the third step was considered for those who were 25 years of age. Data were collected from individuals who agreed to participate and completed inform consent forms. The software features enabled us to analyze non–participation in each of study steps.”

*Source: Djalalinia, S et al., Protocol Design for Large–Scale Cross–Sectional Studies of Surveillance of Risk Factors of Non–Communicable Diseases in Iran: STEPs 2016. September 2017. Archives of Iranian Medicine, Volume 20, Number 9, pages 608-616*

# **Iraq STEPS 2015**

“A cross‐sectional community based survey covering 15 governorates in Iraq.  A Multi-stage cluster sampling technique was depended to select the minimum representative sample size to estimate the prevalence of the risk factors of noncommunicable disease through direct interview, physical examination and laboratory examination of blood samples of study participants. A total of 412 clusters were randomly selected each contain ten households. One subject from each household was randomly selected using KISH table to participate in the survey with a total sample size of 4120. Primary sampling units: The Sample was designed to provide estimates on a number of indicators on the situation of Noncommunicable diseases risk factors in Iraq at the national level. A national based rather than a governorate based sample is selected. A multi stage cluster sampling was used with stratification to urban and rural areas. Primary sampling units (PSUs) were the blocks, which consisted of 70 households or more before selection.”

*Source: Noncommunicable Diseases Risk Factors STEPS Survey Iraq 2015. Available at: https://www.who.int/ncds/surveillance/steps/iraq/en/.*

# **Jordan STEPS 2019**

“A national cross-sectional survey was conducted adopting a two-stage stratified-cluster sampling design. The margin error was (5%) and the confidence level was set at 95%. The Jordan Population and Housing Census 2015 was used as a sampling frame for Jordanians. A sample of 3000 households was randomly drawn to represent the Jordanian population. It was designed in a probability proportional to size (PPS) way to provide valid and reliable survey estimates across the entire Kingdom of Jordan - rural and urban areas, the twelve governorates and the smaller communities within.

The sample also ensured reliable estimates in terms of geographical distribution, where Jordan was divided into three regions; north, centre, and south, also at governorate level. The north of Jordan covered Ajloun, Irbid, Jerash, and Mafraq, the centre region covered Amman, Balqa, Madaba, and Zarqa, and the south region covered Aqaba, Karak, Ma’an, and Tafieleh. Furthermore, each governorate was subdivided into area units called census blocks, which were the Primary Sampling Units (PSU-Blocks) for this survey (on average a PSU comprises 50-70 households).

The PSU-Blocks were then regrouped to form clusters. From each PSU, eight households were randomly drawn with an equal probability systematic selection. A household was defined as a group of people living in the same dwelling space who eat meals together, acknowledging the authority of a man or a woman as the head of the household. After the household selection and obtaining the permission of household residents to participate in the survey, all the eligible household members were entered into the STEPs program, which ran a random selection to choose one member from each household. The sample size from each governorate was proportionate to the population’s size (table,2).

Sampling of Syrians

Sampling of Syrian refugees took place in four governorates; Amman, Zarqa, Irbid and Mafraq, where over than 90% of Syrian refugees reside in Jordan. The sample size was proportional to the population size in each of the governorate, i.e., the bigger the population, the bigger the sample size selected (table, 3). To update the sampling frame for Syrians, a prelisting of households took place, where Syrian households were visited and entered a listing frame (the same DOS listing questionnaire was used). This sampling frame enabled weight calculation and the selection of the final Syrians sample. This was followed by determining the sampling blocks (PSU) which were 375, then from each block, 8 households were randomly selected to fulfill the pre-calculated sample size of 3000.“

*Source: Department of Public Health, Ministry of Health (2020). Non-communicable disease Risk Factors: Jordan STEPS Survey 2019*

**Kazakhstan Household Survey Health Module 2012**

“The Kazakhstan Household Health Survey (KHHS) was conducted in 2012. A nationally representative multi-stage sampling approach was used. First, 452 Census Control Areas (CCA, mean size 1200 inhabitants per area) were randomly selected from a complete list of all CCAs covering the entire country; the list was provided by the Committee of Statistics at the Ministry of National Economy in Kazakhstan. The target sample was allocated to 14 Oblasts (regions) and two major cities (Astana city, the capital of Kazakhstan, and Almaty city) proportional to the region or city population size with small adjustments. Second, 13,560 households were randomly selected from the chosen CCAs. The target population of the survey was all residential households with at least one resident aged 15 years and over. In each household, one eligible respondent completed the survey. Data were collected by trained interviewers using computer assisted personal interviews (CAPI).”

*Source: Pasted verbatim from an email exchange with the study team.*

**Kenya STEPS 2015**

“The 2015 Kenya STEPs survey was a national cross-sectional household survey designed to provide estimates for indicators on risk factors for non-communicable diseases for persons age 18 – 69 years. The sample was designed with a sample size of 6,000 individuals to allow national estimates by sex (male and female) and residence (urban and rural areas). The survey used the fifth National Sample Surveys and Evaluation Programme (NASSEP V) master sample frame that was developed and maintained by KNBS. The frame was developed using the Enumeration Areas (EAs) generated from the 2009 Kenya Population and Housing Census to form 5,360 clusters split into four equal sub-samples. A three-stage cluster sample design was adopted for the survey involving selection of clusters, households and eligible individuals. In the first stage, 200 clusters (100 urban and 100 rural) were selected from one sub-sample of NASSEP V frame. A uniform sample of 30 households from the listed households in each cluster was selected in the second stage of sampling. The last stage of sampling was done using Personal Digital Assistants (PDAs) at the time of survey, where one individual was randomly selected from all eligible listed household members using a programmed KISH method of sampling.”

*Source: WHO: Kenya STEPwise Survey for Non Communicable Diseases Risk Factors 2015 Report. Available at:* [*http://www.who.int/chp/steps/Kenya_2015_STEPS_Report.pdf?ua=1*](http://www.who.int/chp/steps/Kenya_2015_STEPS_Report.pdf?ua=1)*.*

# **Kiribati STEPS 2015**

“As STEPS is intended to be nationally representative, a multi-stage cluster sampling method was used. The STEPS sampling spreadsheet was completed using the most recent census information (2012). The sample was selected in two stages assuming no replacement. At the first stage, a sample of Enumeration Areas (Islands and villages) from each stratum using probability proportional to size (PPS) sampling was selected. In the second stage, a fixed number of households from each selected Enumeration Area using systematic sampling was selected. The third stage of sampling selection was done at the household level using the KISH method. The sampling identified that data collection would be needed on the following islands: Makin, Butaritari, Marakei, Abaiang, North Tarawa, South Tarawa,Betio, Maiana, Abemama, Kuria, Aranuka, Nonouti, Tabiteuea North, Tabiteuea South, Arorae, Tabuaeran and Kiritimati.”

*Source: Kiribati NCD Risk Factors STEPS Report 2015-2016. Available at: https://www.who.int/ncds/surveillance/steps/kiribati/en/.*

# **Kyrgyzstan STEPS 2013**

“A multi-stage cluster sample of households. One individual within the age range of the survey was selected per household.”

*Source: NCD Microdata Repository. Study Description. Available at: https://extranet.who.int/ncdsmicrodata/index.php/catalog/271/study-description#page=sampling&tab=study-desc*

# **Lao People's Democratic Republic STEPS 2013**

“A multi-stage cluster sample of households. One individual within the age range of the survey was selected per household.”

*Source: NCD Microdata Repository. Study Description. Available at: https://extranet.who.int/ncdsmicrodata/index.php/catalog/588/study-description#page=sampling&tab=study-desc*

**Liberia STEPS 2011**

“Random multi-cluster sampling method was used to collect data during this survey in 5 of the 15 counties of Liberia with the district serving as the primary sampling unit. Different sampling frames were designed and used at the district (Primary Sampling Unit-PSU), Chiefdoms (Secondary Sampling Unit-SSU) and household levels. Households listing generated from the 2008 National Population Census was used, and in each household, the list of individuals’ resident was obtained and the Kish Method was used. Kish Method is a household sampling technique developed by WHO for STEPS. The field team selected households by using nutrition sampling method (throwing a pencil to get a selected direction). When the household enumeration sampling point is established, the interviewer counts all the households and using interval sample to get the household number. In each household, one person was selected using the Kish method.”

# *Source: WHO: The Final Report on the Liberia STEPS Survey 2011. Available at:* [*http://www.who.int/chp/steps/Liberia_2011_STEPS_Report.pdf?ua=1*](http://www.who.int/chp/steps/Liberia_2011_STEPS_Report.pdf?ua=1)*.*

# **Lebanon STEPS 2017**

“A national cross-sectional survey adopting a two-stage cluster sampling design was conducted for Steps 1, 2 and 3. The sampling frames references used were the population distribution in Lebanon 2014, retrieved from the Central Administration for Statistics (CAS) and the Syrian population distribution data 2015, retrieved from UNHCR. 144 clusters were selected for the Lebanese sample and 144 clusters for the Syrian sample. The Primary Sampling Units (PSUs) were cadastral areas (cadasters) and the Secondary Sampling Units (SSUs) were the households. Twenty participants were recruited from each cluster. The latest available population estimates (cadastral data) were used, to randomly recruit PSUs by Probability Proportionate to Size (PPS). To account for the issue of the variability in the cadasters’ sizes, very small cadasters (<200 individuals) were combined with neighboring PSUs before selecting the sample, to enhance the likelihood of finding 20 target participants. On the other hand, cadasters with a large population size that were guaranteed to be sampled at least twice were handled as strata and each stratum were assigned a fixed number of random starting points based on how often it was selected with certainty. This was done using satellite images divided into grids, previously obtained from the Centers for Disease Control and Prevention (CDC)1 for all Lebanese cadasters. For the Lebanese sample, the research team relied on the standard Expanded Program for Immunization (EPI) method for a systematic random selection of the households. Accordingly, within each selected PSU, households were identified using a systematic random approach following the WHO-UNICEF-EPI cluster method. The fieldworkers started with the highest floor on the right side of a building. If the household hosted an eligible participant, they proceeded with data collection, if not, they visited a second household which is selected by skipping 5 households. If during sampling, non-Lebanese households were selected, the fieldworker skipped them in a straight line until a Lebanese household was identified. This method has been previously used for national surveys in Lebanon. One participant was randomly selected within each household, using the eSTEPS application. Households were chosen until the target of 20 participants was reached. The PSUs for the Syrian refugees’ sample were identified, using the most recent available refugee estimates to randomly recruit PSUs by PPS. The same measures aforementioned were done to account for the variation in the cadasters’ sizes. The WHO-UNICEF- EPI cluster method was employed to select households. The fieldworkers targeted Syrian households; accordingly, when during sampling, non-Syrian households were selected, the fieldworker skipped them in a straight line until a Syrian household was identified. One participant was randomly selected within each household, using the eSTEPS application.  For both samples, following STEPS’ team recommendations, sampling of participants was done without replacement, i.e. once a person was selected that person was not replaced with another one. Efforts were made to include all selected households. If the house was unoccupied at the time of the visit or if an adult was not available for an interview at the time of the visit, that house was revisited up to 4 times, with different visiting times. The number of refusals and nonresponses was recorded.”

*Source: WHO Stepwise Approach for Non-Communicable Diseases Risk Factor Surveillance Lebanon 2016-2017. Available at: https://www.who.int/ncds/surveillance/steps/lebanon/en/.*

# **Malawi STEPS 2017**

“Stage 1: Selection of enumeration areas (EAs):

*Sampling frame:*

Malawi is divided into twenty-eight districts. In turn, each district is subdivided into smaller administrative units called traditional authorities (TAs). Each administrative unit is sub-divided into EAs by the National Statistical Office (NSO). EAs are classified as urban or rural. Each EA has a sketch map drawn by NSO, which shows the EA boundaries, location of buildings, and other landmarks. A list of EAs obtained from NSO was used as a sampling frame for random selection of EAs as described below. ^1^

*Number of enumeration areas to be selected*

In accordance with WHO STEPS Manual, the recommended number of participants to be selected at each primary sampling unit (in our case in each EA) is 20. Given that the estimated required sample size was 5,088, the total number of EAs selected was 5,088/20=255. ^1^

*Sampling method for EA selection:*

Probability Proportional to Size (PPS) sampling method was used to randomly select the 255 EAs from the whole country as follows:

- The EAs in Microsoft Excel was first sorted in descending order of population (largest to the smallest).
- Then the total population of all EAs in Malawi was calculated.
- A column of cumulative total population of EAs was created.
- Then the sampling interval was calculated by dividing total population by 255 (total number of EAs to be selected).
- Random number (the seed) was generated by computer in the Excel using the formula RANDBETWEEN (sampling interval).
- The EA whose cumulative total contained the seed was the first to be selected.
- The 2nd, 3rd, up to 255th EA was selected systematically by adding the random number to the sampling interval.

Stage 2: Selection of households

The EA sampling frame obtained from NSO had information on the total number of households in each EA. Twenty households were selected from each EA. The sampling interval for household selection in each EA was therefore determined by dividing the total number of households in the EA by 20. Systematic sampling method (every nth household) was then used to randomly select the required 20 households. ^1^

Stage 3: Selection of eligible participants at household level

Only one eligible participant (an adult aged 18-69 years) in the selected households was enrolled in the survey. In households with more than one eligible participant, participants were randomly selected using an Android device.^”^

*Source: Malawi Ministry of Health. Malawi National STEPwise Survey for Non-Communicable Diseases Risk Factors 2017 Report*

# **Marshall Islands STEPS 2017**

# “Stage 1: Households were identified at random according to geographical stratification in Majuro and Ebeye. The country was stratified into two major groups, Urban (Majuro and Ebeye) and Rural (all outer islands). In Majuro and Ebeye, household cluster sampling was used to randomly select households in these areas.

# Stage 2: In Majuro and Ebeye, one individual was selected at random from each household using the KISH table method. All adults in Kili, Arno, Wotje, and Jabwor, Jaluit atolls were included in the sample because the adult populations are about 200 each on these atolls.”

# *Source: Ritz S., Cash, H. (2018) Republic of the Marshall Islands Hybrid Survey: Final report.*

#

# **Mexico ENSANUT 2018**

The Primary Sampling Units (PSU) were selected from INEGI's 2012 Master Sample of Dwellings (MMV 2012), which was selected from the cartographic and sociodemographic information of the 2010 Population and Housing Census. The 2012 MMV is the basis for selecting the samples for the housing surveys conducted by INEGI. The MMV 2012 and Ensanut 2018-19 PSUs are classified by two stratification criteria: 1) size of the locality and 2) sociodemographic conditions of the dwellings. There are four sociodemographic strata that were constructed by summarizing 34 indicators that describe the physical characteristics and equipment of the dwellings, as well as, sociodemographic characteristics of the inhabitants of the dwellings. On the other hand, there are three strata of locality size: rural (localities with less than 2,500 inhabitants), high urban (cities with 100,000 or more inhabitants) and urban complement (localities with a number of inhabitants between 2,500 and less than 100,000 inhabitants).

Once the PSUs and strata were constructed, the PSUs for the 2018-19 Ensanut were selected in two stages: first, INEGI selected a master sample of PSUs with probability proportional to their number of dwellings in the year 2012, then, for the 2018-19 Ensanut, a subsample of PSUs with equal probability was selected within each stratum. Finally, in each PSU, dwellings were selected with equal probability; on average, five dwellings were selected in each PSU of the high urban stratum and 20 dwellings in the PSUs of the rural and urban complement strata.^2^

*Source: translated from:* Shamah-Levy T, Vielma-Orozco E, Heredia-Hernández O, Romero-Martínez M, Mojica-Cuevas J, Cuevas-Nasu L, Santaella-Castell JA, Rivera-Dommarco J. Encuesta Nacional de Salud y Nutrición 2018-19: Resultados Nacionales. Cuernavaca, México: Instituto Nacional de Salud Pública, 2020.

# **Moldova STEPS 2013**

“A two-stage cluster sampling procedure was carried out to select randomly participants from among the target population. Cluster sectors from the 2004 Moldova Population Census were used as a basic unit. Given the differences in lifestyle and disease status between populations in urban and rural areas, the target population was stratified into urban and rural areas of residence for the STEPS survey. At the first stage, within each stratum, primary sampling units (PSUs) (enumeration areas (EAs)) were selected systematically with probability proportional to the 2004 Population Census EAs (measure of size equal to the number of population in the EAs, provided by the census). Before selection, the census sectors were sorted geographically from north to south within each stratum, in order to ensure additional implicit stratification according to geographical criteria. A total of 400 clusters representing 400 EAs were selected from the 10 991 census EAs. These probabilistically selected clusters were used also in Moldova’s DHS conducted in 2005, and the Multiple Indicator Cluster Surveys (MICS) conducted in 2012. Cartographic materials from the Population Census conducted in Moldova in 2004 were not available, thus it was not possible to use them for the STEPS survey. Therefore, for the first stage the probabilistic samples from the abovementioned surveys were used. Out of the 400 selected clusters, 167 were rural and 233 were urban. The distribution of the sample of 400 PSUs (EAs) for the DHS/MICS surveys was inversely proportional to the number of population within each stratum, taking into account that the response rate is lower in urban areas than rural owing to the smaller average size of the households in urban areas compared with rural areas. Thus, disproportional allocation with oversampling for urban areas was applied in the STEPS survey. A final weighting adjustment procedure was carried out to enable estimates at national and urban/rural levels. At the second stage, 15 households (secondary sampling units (SSUs)) were selected within each of the 400 PSUs. From the updated list of households used for the MICS 2012 survey, 15 households were selected randomly per cluster, using the Microsoft Excel® random sample tool. A total of 6000 individuals were selected from among the 400 clusters.

The Kish method  was applied for the random selection of one individual aged 18–69 years from each household.”

*Source: Prevalence of Noncommunicable Disease Risk Factors in the Republic of Moldova STEPS 2013. Available at: https://www.who.int/ncds/surveillance/steps/moldova/en/.*

# **Mongolia STEPS 2013**

“The survey was designed to cover all geographical areas of Mongolia, and a multi stage stratified sampling process was carried out to randomly select participants from the target population. Given the urban vs. rural differences in lifestyle and disease status, the target population was stratified into urban and rural areas and the sample was drawn proportionally based on the target population in each area. Ulaanbaatar, Darkhan and Erdenet cities represented urban areas, while the remaining aimags and soums represented rural areas. Primary units for Ulaanbaatar, Darkhan and Erdenet cities were khoroos, whereas soums served as primary units for rural areas. The same principle used in the previous STEPS surveys in 2005 and 2009 was applied for sampling unit selections for each stage. From each selected household at the tertiary units of multi-stage cluster sampling in both urban and rural areas, only one individual aged 15-64 years old was randomly selected. The survey covered a total of 65 cluster sampling units. These units included randomly selected individuals from 32 soums in 21 rural aimags and 33 khoroos in Ulaanbaatar, Darkhan and Erdenet cities. In order to be able to compare the survey results and findings by urban and rural areas, we conducted sampling based on the principles to select approximately similar numbers of participants from both urban and rural areas.”

*Source: Third national STEPS Survey on the Prevalence of Noncommunicable Disease and Injury Risk Factors-2013. Available at: https://www.who.int/ncds/surveillance/steps/mongolia/en/.*

# **Mongolia STEPS 2019**

“A multistage stratified sampling design was used to produce representative data for that age range in Mongolia. A total of 6654 adults participated in the survey.

6654 people representing the population of Mongolia aged 15-69 were involved to the 1st and 2nd steps of the survey, and 6497 people were in the 3rd step of the survey. As 98.1% of sample population were covered.

Analysis weights were calculated by taking the inverse of the probability of selection of each participant. These weights were adjusted for differences in the age-sex composition of the sample population as compared to the target population.

Different weight variables are available per Step:

wStep1 - for interview data

wStep2 - for physical measures

wStep3 - for biochemical measures

This allows for differences in the weight calculation for each Step of the survey as the age-sex composition of the respondents to each Step can differ slightly due to refusal or drop out. Additionally, some countries perform subsampling for Step 2 and/or Step 3. When no subsampling is done and response rates do not differ across Steps of the survey, the 3 weight variables will be the same.

*Source: NCD Microdata Repository. Study Description. Available at: https://extranet.who.int/ncdsmicrodata/index.php/catalog/836/study-description#metadata-data_access*

# **Morocco STEPS 2017**

“First stage (circulation of UP primary units): 244 (158 Urban Primary Units: UPU and 86 Rural Primary Units: UPR) primary units were drawn from the 4,500 UP constituting the master sample. Each UP is a geographic area with clear and identifiable boundaries on the ground and comprising on average 300 households. Second stage (drawing of secondary units (US) or clusters): When developing the master sample, the primary units were subjected to a cartographic division into zones of 50 households each. As a result, a UP has 6 secondary units on average. For the purpose of the STEPS survey, a single secondary unit or cluster was selected at the level of each sample UP. Each UP contains on average 6 clusters. Third stage (household circulation): Based on the cartographic sketch of the secondary unit (cluster) and starting from the starting point mentioned on the US limit sheet, and in a clockwise direction the interviewer draws the households to be surveyed by applying a step of two households.In each household, a random sampling of all individuals meeting the selection criteria was made, and a single participant is drawn at random in each household via the e-Steps application.”

*Source, translated from: Enquête Nationale sur les Facteurs de Risque communs des Maladies Non Transmissibles 2017 – 2018 : Rapport. Available at: https://www.who.int/ncds/surveillance/steps/morocco/en/.*

# **Mozambique STEPS 2014-2015**

“The sample was designed based on data from the 2007 census [10], to be representative at the national and provincial levels, and according to the residence in urban or rural areas; the homeless and people living in collective residential institutions (e.g. hotels, hospitals, military facilities) were not eligible. Participants were selected in three stages. The first stage included the selection of 120 primary sampling units (geographical units including 400–600 households in the urban areas and 400–500 households in the rural areas), with probability proportional to the number of households, stratified according to province, urban or rural areas and socioeconomic strata; the latter were considered only in cities with more than 20 000 households. The second stage included the random selection of one enumeration area (geographical unit including 100–150 households in the urban areas and 80–100 households in the rural areas) within each primary sampling unit, corresponding to a total of 120 clusters. The third stage included an update of the list of households in each enumeration area selected, followed by random and systematic selection of 24 households. Within each selected household, all dwellers aged 15–64 years were listed and a maximum of two were selected, one aged 15–44 years and one aged 45–64 years, whenever available; whenever there was more than one household member in each of these age groups, only one per group was randomly selected, using a Kish selection grid.”

*Source: Jessen, Neusa, Albertino Damasceno, Carla Silva-Matos, Edite Tuzine, Tavares Madede, Raquel Mahoque, Patrícia Padrão, Francisco Mbofana, Jorge Polónia, and Nuno Lunet. "Hypertension in Mozambique: trends between 2005 and 2015." Journal of hypertension 36, no. 4 (2018): 779-784.*

# **Myanmar STEPS 2014**

“To achieve a nationally representative sample, a multi-stage sampling method was used to select townships, wards and villages, households and eligible participants at each of the selected households.  Stage 1: Selection of primary sampling units (PSUs)  Administratively, Myanmar is divided into 330 townships. A township is subdivided into wards for urban settings and village tracts and then villages for rural settings. The list of townships has been used as the sampling frame at the first stage of sampling. Townships form the Primary Sampling Units (PSUs). Out of the total 330 PSUs, 52 PSUs were selected using Probability Proportionate to Size of population in each PSU (PPS). Stage 2: Selection of Secondary Sampling Units (SSUs) From each selected PSU (township), 6 SSUs (wards and villages) were chosen using probability proportionate to population size, totaling 312 SSUs for the whole country.   Stage 3: Selection of eligible participants at household level From each selected SSU (ward/village), 30 households were selected using systematic random sampling. The sampling frame for this sampling is the list of households with unique identification number (ID) developed from a recent listing of households available from the Basic Health Staff.  Stage 4: Selection of eligible participants at household level One eligible participant (aged between 25 and 64 years) in the selected households was recruited for the survey. The Kish sampling method was used to randomly select one eligible member of the household. Using the Kish Method, eligible participants (adults aged 25 to 64 years) in each household were ranked in order of decreasing age, starting with males then females, then randomly selected using the automated program for Kish selection in the handheld PDA.  Each PSU (township) was estimated to contribute 180 participants, totaling 9,360 participants for 52 selected townships for the whole country. In actual study, the total sample size was 8757 participants.”

*Source: Report on National Survey of Diabetes Mellitus and Risk Factors of Noncommunicable Diseases in Myanmar (2014).  Available at: https://www.who.int/ncds/surveillance/steps/myanmar/en/.*

**Namibia Demographic and Health Survey 2013**

“The sample for the 2013 NDHS was a stratified sample selected in two stages. In the first stage, 554 EAs were selected with a stratified probability proportional to size within the sampling frame. The EA size is the number of households residing in the EA and recorded in the 2011 NPHC. Stratification was achieved by separating each region into urban and rural areas. Therefore, the 13 regions were stratified into 26 sampling strata: 13 rural strata, and 13 urban strata. Samples were selected independently in each stratum, with a predetermined number of EAs selected as shown in Table A.3. Implicit stratification with proportional allocation was achieved at each of the lower administrative unit levels by sorting the sampling frame before the sample selection. Sorting was done according to the constituency and the EA code within a sampling stratum, and by using a probability proportional-to-size selection procedure.

After the selection of EAs and before the main survey, a household listing operation was carried out in all selected EAs, and the resulting lists of households served as a sampling frame for the selection of households in the second stage. Some of the selected EAs may large. To limit the amount of work done to list each household, selected EAs with more than 200 households were segmented by the listing team in the field before the household listing. Only one segment was selected for the survey, with probability proportional to the segment size. Household listing was conducted only in the selected segment (see detailed instructions for segmentation in the DHS Manual for Household Listing). So a 2013 NDHS cluster is either an EA or a segment of an EA. In the second-stage selection, a fixed number of 20 households was selected in every urban cluster and rural cluster, by equal probability systematic sampling. A spreadsheet indicating the selected household numbers for each cluster was prepared. The survey interviewers interviewed only the pre-selected households. To prevent bias, no replacements and no changes of the pre-selected households were allowed in the implementing stages. In half of the selected households where there was no male survey, all women age 15-49 were interviewed; in the other half of the selected households where there was a male survey, all males and females age 15-64 were interviewed.”

# *Source: The Nambia Ministry of Health and Social Services (MoHSS) and ICF International. 2014. The Namibia Demographic and Health Survey 2013. Windhoek, Namibia, and Rockville, Maryland, USA: MoHSS and ICF International*

# **Nauru STEPS 2015**

“As STEPS is intended to be nationally representative, a simple random samples of individuals was identified, based on the most recent census survey.”

*Source: NCD Microdata Repository. Study Description. Available at: https://extranet.who.int/ncdsmicrodata/index.php/catalog/637/study-description*

# **Niger STEPS 2007**

“In the first stage, the EAs were taken from those delimited in the Third General Census of Population and Housing. The EAs were drawn up systematically and independently in each stratum. For each sample, the cumulative size of the population of each stratum was first calculated in the sampling frame. The Polling interval was then calculated as follows: I =M/a, rounded to the nearest whole, where M is the population of the stratum according to the sampling frame and the number of EAs to be drawn in the stratum. The series of survey numbers R, R + I, R + 2I, R + 3I, etc. was calculated, where R is the random number between 1 and I. Each survey number was then compared to the cumulative headcount column. The first EA that was drawn is the first EA on the list whose cumulative workforce is equal to or greater than the first survey number. The second EA that was drawn is the one that follows (after the first EA that has been drawn) whose cumulative number is equal to or greater than the second survey number, and so on. (...) 30 households were systematically drawn by EA. The step is therefore equal to the number of households in the EA divided by 304. In the household, a person meeting the age criterion was interviewed, that is to say between the ages of 15 and 64. In the event that the quota for the 55-64 age group is difficult to reach, two participants have been selected in households where there is an adult aged 55 to 65, this in accordance with the Kish method.”

*Source, translated from: Mesure des facteurs de risque des maladies non transmissibles Au Niger (Approche Step"wise" de l’OMS). Available at: https://www.who.int/ncds/surveillance/steps/niger/en/*

# **Palau STEPS 2011**

“The required sample size was calculated as 2,807 individuals on a margin of error of 0.05, an anticipated response rate of 80% power to detect statistically significant differences between eight age/sex groups. Accordingly, from the 2,807 selected households 2,212 individuals aged 25-64 years participated in STEPs giving an overall response rate of 79% (response rates for parts of STEP2 and 3 were lower).

The survey used a cluster-based sampling design where the primary sampling unit was enumeration area (EA) and the secondary sampling unit was households. The 16 states in Palau were included in the sampling frame. Seventy five (75) EAs were selected using probability proportional to size (PPS). There were 2,807 households that were randomly selected from the total number of 3,976 households.”

*Source: Department of Public Health, Ministry of Health (2020). Non-communicable disease Risk Factors: Palau STEPS Survey 2011-13, Ngerulmud*

# **Paraguay STEPS 2011**

“A cross-sectional study was carried out, with a three-stage probabilistic sample design. no replacement. For the selection of the dwellings and the people to be surveyed, the following Next steps:

a. The cartography of the 2002 Census was used for the formation of 250 Units Primary Sampling (UPM). In urban areas each PSU contained on average 30 homes and 36 in rural areas. The conformation of the UPM preserves the divisions in Enumeration Areas used in the 2002 Census.

b. The Supervisor of each team verified and updated the UPM area and within from it, 2 compacts of 6 consecutive dwellings were formed in each one, with random start, according to a table provided for each PSU. The dwellings were the Secondary Sampling Unit (USM).

c. In the dwellings, while the survey was being carried out, all the household members and, among members 15 to 74 years of age who live in the same, we proceeded to select one of them (Tertiary Unit of Sampling -UTM-), randomly using the Kish method.”

*Source: Ministerio de Salud Pública y Bienestar Social. Primera Encuesta Nacional de Factores de Riesgo de Enfermedades No Transmisibles. 2012.*

# **Peru DHS 2012**

“In order to provide information that allows timely and reliable estimation of the indicators identified in the Strategic Programs for the period 2012 to 2014, the sample has been designed for the ENDES Continuous Demographic and Family Health Survey 2012 – 2014, before the completion of the Sample Teacher 2009-2011. This is a probabilistic master sample that was selected in two stages: the selection of clusters in the first stage and selection of dwellings in the second stage. The sample Master consists of 2 852 clusters, randomly distributed in four subsamples of conglomerates to be executed in three consecutive years. Each year of study has 1 426 clusters, distributed proportionally in the urban and rural areas of each department. For ENDES 2012, the number of clusters per department was around 53, with the exception of Lima where 200 clusters were selected. The total sample allows solid estimates of indicators for households and for women between 15-49 years of age for a number of important variables at an annual level, in each of the 24 departments and natural region of the country (Coast, Sierra, Selva and Metropolitan Lima). For the total sample, the number of dwellings was 28,376, of which only 27,488 were interviewed. In the households interviewed, a total of 24,552 eligible women were found, from whom the a total of 23,888 complete interviews. The selected sample represents the entire population.”

*Source: translated from: Instituto Nacional de Estadística e Informática. Perú: encuesta demográfica y de salud familiar ENDES 2014. Perú: Instituto Nacional de Estadística e Informática; 2015*

**Romania SEPHAR II**

“Sampling was performed by a multi-stratified procedure, leading to the selection of a representative sample of 1942 adults. Subject selection followed the principle of equality of chances of being enrolled in the study, regardless of the size of the place of residency.

Stratification criteria for sample selection were:

- territorial regions (Romania's territory was divided into 7 regions plus the capital city Bucharest, based on the National Statistics Institute recommendations: the North-East region, the South-East region, the South region, the South-West region, the West region, the North-West region, the Central region and the Bucharest region);
- locality type (cities with over 200 000 inhabitants, cities with 50 000–200 000 inhabitants, cities with less than 50 000 inhabitants, Commune);
- gender (male and female);
- age groups (18–24 years, 25–34 years, 35–44 years, 45–54 years, 55–64 years, 65–80 years).

In the first stage of selection, the adult population weighted average was calculated for each region and each district, and, based on this, the number of adult persons from each region/district was calculated from the working sample of 1942 subjects.

In the second stage of selection, the number of localities of a certain size from which the subjects were later selected was established for each district. This number was directly proportional to the population in the respective district. A random selection of a certain locality in a certain category was done using a computer software (generation of random numbers). The selected localities represent the interview centers where the study was to take place. The weighted average of the specific locality population in the district was calculated, and, based on this, the number of people selected to participate in the study.

The third stage of selection consisted of distribution by gender of adult people selected from each locality, using Romania's population gender distribution according to the 2002 census (F : M = 51.25% vs. 48.75%) and the fourth stage of selection consisted of distribution by age of male and female adult people selected from each locality, using Romania's population age distribution according to the 2002 census.”

*Source: Dorobantu M, Tautu OF, Darabont R, Ghiorghe S, Badila E, Dana M, Dobreanu M, Baila I, Rutkowski M, Zdrojewski T. Objectives and methodology of Romanian SEPHAR II Survey. Project for comparing the prevalence and control of cardiovascular risk factors in two EastEuropean countries: Romania and Poland. Arch Med Sci. 2015 Aug 12;11(4):715-23.*

**Russian Federation Study on global AGEing and adult health, Wave 1 (2007-2008)**

“The SAGE Russian national sample was constructed using data from two sources:

1. The sample for the 2003 World Health Survey (WHS)

2. The 2002 All-Russia Population Census.

In constructing the SAGE Russian national sample, efforts were made to ensure even representation across administrative units. The largest administrative unit in the Russian Federation is the Federal District (FD). In 2007, there were seven FDs: Central, Southern, Northwestern, Urals, Volga, Siberian, and Far Eastern. Each FD is made up of federal subjects, administrative divisions which have varying levels of autonomy, but equal representation in the federal government; these include republics, krais, oblasts, federal cities, autonomous oblasts and autonomous okrugs. In 2007, there were 86 federal subjects. For the purpose of SAGE, federal subjects with particularly low population densities (making up 0.2% of the total population of Russia) were excluded: these included the Yamal-Nenets, Taimyr, Evenki, Koryak, and Chutkhotka autonomous okrugs, the republic of Sakha (Yakutia), Khabarovsk krai, and Magadan oblast.

2.1.1 First stage

The first stage of sample design was the definition of strata for selection. The sample was initially stratified by FDs, according to the distribution of population

2.1.2 Second stage

The second stage of the sample design was selection of primary sampling units (PSUs), mainly according to the data from the 2002 Russian Census. For the selection of PSUs, first, all households which had participated in the WHS and which had a member aged 50-plus were listed again, as well as some WHS households with a member aged 18-49. These households were drawn from the three FDs that were included in the WHS: Central, Northwestern, and Volga.

Next, PSUs were selected for the remaining four FDs (Southern, Siberian, Urals and Far Eastern). Within FDs, administrative and territorial formations (ATF) constituted the primary sampling units (PSU). The population distribution within the four districts was used to determine the number of sample localities in each district, which were then weighted to reflect their representation in the four districts.

A computer program (according to the PPS method) was then used to select specific settlements in each district from the total number of ATFs, according to census data. ATFs were selected randomly and proportionally to the size of federal districts’ population. This resulted in 39 ATFs from the Southern FD, 34 from the Siberian FD, 20 from the Urals FD and 7 from the Far Eastern FD. From each ATF, households, which constituted the study’s secondary sampling units (SSU), were chosen at random, using a special formula for each territory sample. The probability of being including in the sample was equal for all households (0.00247). Between 1 and 551 households were selected from each ATF. Address lists for all selected households (including house and apartment numbers) were compiled with the help of out-patient clinic staff. In each territory, the sample was based on a household listing and enumeration, randomly selecting houses/ apartments until the desired sample size was reached. All members of each household selected for the survey sample were enumerated on the household roster and all eligible people aged 50-plus were invited to participate in the survey. If a household had at least one person aged 50 or older, then that household was included in the 50-plus sample. In the remaining households (that is, with no member aged 50 or older) one respondent aged 18–49 was randomly selected using Kish tables (Kish, 1965; Kish, 1987).

In selected households, the individuals eligible for interview formed the ultimate sampling unit. The total sample size of individuals was targeted to be 1000 people in the age group 18–49 years old and 5000 people aged 50 or older.

2.1.3 Stratification and allocation of enumeration areas

From a total of 288 enumeration areas, 176 ATFs were visited: the coverage was 61.1% of the targeted ATFs, with the highest percent of visited enumeration areas in the Southern FD (71.8%). Of 7,200 eligible households, 4,644 HHs were included in the final sample and visited (both in urban and rural territories. 1,407 of them took part in the WHS survey.”

# *Source: Study on global AGEing and adult health (SAGE) Wave 1 Russian Federation National Report National Research Institute of Public Health, Russian Academy of Medical Sciences (RAMS). Study Report December 2013*

# **Rwanda STEPS 2012**

“Multistage cluster sampling was used to select these participants from the population based on information from the last census. The three levels of clustering were: 1. Random selection of a statistical enumeration area (as defined by NISR) 2. Random selection of a household within the enumeration area 3. Random selection of an individual within the household. Selection of Enumeration Areas: Administratively, Rwanda is divided into thirty districts. In turn, each district is subdivided into sectors. Each sector is sub-divided into cells and then into villages. Villages are synonymous with enumeration area’s (EAs) in Rwanda and there are a total of 14,953 EAs in Rwanda. A total of 180 EA’s (or 1.2%) were randomly selected from this total using a probability proportional to size method that gives those EA’s with more people living in them a higher chance of being selected. In this way, the representativeness of the selected EAs is maximized.  Selection of households: Forty households were randomly selected from within each of the selected EAs from a list of households supplied by NISR based on the most recent census.  Selection of eligible participants:  One eligible participant (an adult aged 15-64 years) was randomly selected from within each household using the Kish sampling method which is built into the PDAs used in the survey.”

*Source: Rwanda Non-communicable Diseases Risk Factors Report November 2015. Available at: https://www.who.int/ncds/surveillance/steps/rwanda/en/.*

# **Samoa STEPS 2013**

“The STEPS survey was a population-based survey of adults aged 18-64. A multi-stage, cluster sample design was used to produce representative data for that age range in Samoa. A total of 1766 adults participated in the survey. The overall response rate was 64%.”

*Source: Samoa STEPS Survey 2013 Fact Sheet. Available at: https://extranet.who.int/ncdsmicrodata/index.php/catalog/707*

# **São Tomé and Principe STEPS 2008**

“The São Tomé and Principe STEPS survey is a survey of the general population, targeting adults aged 25 to 64. A cluster draw was used to produce representative data for this age group in São Tomé and Principe. A total of 2,457 adults participated in the São Tomé and Principe STEPS survey.”

*Source: São Tomé et Principe Enquête STEPS 2008 Note de synthèse. Available at: https://www.who.int/ncds/surveillance/steps/sao_tome_and_principe/en/*

# **São Tomé and Principe STEPS 2019**

“A multi-stage cluster sample of household. One individual within the age range of the survey was selected per household. Target sample size was 2650.”

*Source: Survey description on Microdata repository available at: https://extranet.who.int/ncdsmicrodata/index.php/catalog/893/study-description*

**Seychelles STEPS 2013**

“The survey was performed in a sex and age stratified random sample of all adults aged 25-64 years of Seychelles between October and December 2013 on Mahé and during 2 weeks in February 2014 in the islands of Praslin and La Digue. These three islands account for >98% of the total population of Seychelles. The eligible sample was extracted from the population registry. The survey was attended by 1240 adults, with a participation rate of 73%. Participants were invited to attend the survey on selected days in study centers located in Mahé, Praslin, and La Digue. All the eligible participants who did not attend were actively traced using (telephone, local administration, announcements on radio, etc) and invited to attend the survey. Since participants were randomly selected from the general adult population, findings of the survey can be inferred to the general adult population of Seychelles.”

*Source: National Survey of Noncommunicable Diseases in Seychelles 2013-2014 (Seychelles Heart Study IV): methods and main findings. Available at:* [*http://www.who.int/chp/steps/seychelles/en/*](http://www.who.int/chp/steps/seychelles/en/)*.*

# **Sierra Leone STEPS 2009**

“The multi-stage cluster sampling strategy was used in this study. The CSs as demarcated by Statistics Sierra Leone (SSL) were used as the first set of clusters i.e. primary sampling unit (PSU). Hundred CSs were selected using the probability proportionate to size (PPS) sampling method. The CSs contain several EAs which served as the secondary sampling units. Five hundred and fifty EAs were selected from within the selected CSs by the PPS sampling method. At the tertiary stage, at least ten households were selected from each selected EA by using a simple random technique. Finally, one eligible respondent was selected from the list all eligible respondents within a selected household using the Kish method as describe in

the WHO stepwise approach to chronic diseases surveillance manual.”

*Source: The prevalence of the Common Risk Factors of Non-Communicable Diseases in Sierra Leone. Available at: https://www.who.int/ncds/surveillance/steps/sierra_leone/en/.*

# **Solomon Islands STEPS 2015**

“A multi-stage cluster sample design was used to produce representative data.”

*Source: NCD Microdata Repository. Study Description. Available at: https://extranet.who.int/ncdsmicrodata/index.php/catalog/710/study-description#page=sampling&tab=study-desc*

**South Africa SANHANES 2012**

“The survey applied a multi-stage disproportionate, stratified cluster sampling approach. A total of 1000 census enumeration areas (EAs) from the 2001 population census were selected from a database of 86,000 EAs and mapped in 2007 using aerial photography to create the 2007 HSRC master sample to use as a basis for sampling of households. The selection of EAs was stratified by province and locality type. In the formal urban areas, race was also used as a third stratification variable (based on the predominant race group in the selected EA at the time of the 2001 census). The allocation of EAs to different stratification categories was disproportionate, in other words, over-sampling or over-allocation of EAs occurred in areas that were dominated by Indian, coloured or white race groups to ensure that the minimum required sample size in those smaller race groups were obtained. Based on the HSRC 2007 Master Sample, 500 Enumerator Areas (EAs) representative of the sociodemographic profile of South Africa were identified and a random sample of 20 visiting points (VPs) were randomly selected from each EA, yielding an overall sample of 10 000 VPs. EAs were sampled with probability proportional to the size of the EA using the 2001 census estimate of the number of VPs in the EA database as a measure of size (MOS). One of the tasks of SANHANES-1 was to recruit and establish a cohort of 5 000 households to be followed up over the coming years. The sampling consisted of: Multi-stage disproportionate, stratified cluster sampling approach; 500 EAs within which 20 VPs/households per EA were sampled; Main reporting domains: sex (male, female), age-group (< 2 years, 2–5 years, 6–14 years, 15–24 years, 25–49 years, 50 years and older), race group (black African, white, coloured, Indian), locality type (urban formal, urban informal, rural formal [including commercial farms] and rural informal], and province (Western Cape, Eastern Cape, Northern Cape, Free State, KwaZulu-Natal, North West, Gauteng, Mpumalanga, Limpopo).”

*Source: Human Sciences Research Council. SANHANES: Health and Nutrition. 2015. Available at:* [*http://www*](http://www)*.hsrc.ac.za/en/research-areas/Research_Areas_PHHSI/sanhanes-health-andnutrition*

**South Africa DHS 2016**

“The sampling frame used for the SADHS 2016 is the Statistics South Africa Master Sample Frame (MSF), which was created using Census 2011 enumeration areas (EAs). In the MSF, EAs of manageable size were treated as primary sampling units (PSUs), whereas small neighbouring EAs were pooled together to form new PSUs, and large EAs were split into conceptual PSUs. The frame contains information about the geographic type (urban, traditional, or farm) and the estimated number of residential dwelling units (DUs) in each PSU. The sampling convention used by Stats SA is DUs. One or more households may be located in any given DU; recent surveys have found 1.03 households per DU on average.

Administratively, South Africa is divided into nine provinces. The sample for the SADHS 2016 was designed to provide estimates of key indicators for the country as a whole, for urban and non-urban areas separately, and for each of the nine provinces in South Africa. To ensure that the survey precision is comparable across provinces, PSUs were allocated by a power allocation rather than a proportional allocation. Each province was stratified into urban, farm, and traditional areas, yielding 26 sampling strata.^1^

The SADHS 2016 followed a stratified two-stage sample design with a probability proportional to size sampling of PSUs at the first stage and systematic sampling of DUs at the second stage. The Census 2011 DU count was used as the PSU measure of size. A total of 750 PSUs were selected from the 26 sampling strata, yielding 468 selected PSUs in urban areas, 224 PSUs in traditional areas, and 58 PSUs in farm areas. ^2^

A listing operation was carried out in all selected PSUs from January to March 2016, and the updated lists of DUs served as a sampling frame for the selection of DUs in the second stage. In the second stage of selection, a fixed number of 20 DUs per cluster were selected with systematic selection from the created listing. All households in a selected DU were eligible for interviews.

Some of the selected PSUs were informal, unstructured settlements with no clear identifications of DUs. To ensure listing coverage within each informal, unstructured PSU selected,^3^ segmentation was carried out, with the PSU divided into multiple segments of about 20 DUs each. Only one segment was selected at random for the survey; in segments with 20 DUs or fewer, all DUs in the segment were eligible for the survey. In segments with more than 20 DUs, 20 DUs were randomly selected and were eligible for the survey. A cluster in the SADHS 2016 is therefore either a PSU or a segment of a PSU.

In the remaining half of DUs, all households were eligible for interviews with the Household Questionnaire, and all women and men age 15 and older who were either permanent residents of the selected households or visitors who stayed in the household the night before the survey were eligible for individual interviews and for biomarker collection. Women age 15-49 and men age 15-59 were eligible for the standard individual questionnaire, as well as a South Africa-specific module on adult health; women age 50 and older and men age 60 and older were eligible for a few sections of the individual questionnaire and the adult health module. In addition, children age 0-59 months were eligible for biomarker collection.

Finally, in all households in selected DUs, one woman age 18 and older was selected for a module on domestic violence. In addition, for each child age 0-5 whose biological mother did not live in the household, a guardian was eligible to complete the Caregiver’s Questionnaire.”

Footnote 1 “Western Cape does not have traditional residential geotype PSUs, so only two substrata are applicable.”

Footnote 2 “Four PSUs were dropped from the sample: one was vacant, two were non-accessible due to refusals, and one was an industrial area.”

Footnote 3 “There were 26 informal, unstructured PSUs in the SADHS sample.”

*Source: South Africa Demographic and Health Survey 2016 Report. Available at: https://www.dhsprogram.com/publications/publication-fr337-dhs-final-reports.cfm*

# **Sri Lanka STEPS 2014**

“A multi stage cluster sampling method was used to select a nationally representative sample from the total population. Department of Census and Statistics of Sri Lanka performed the selection of the study sample. Population of each divisional secretariat (DS) divisions as per the preliminary results of the Census done in 2012 was used for sampling. Sri Lanka is administratively divided in to 9 provinces and 25 districts. Each district is divided to Divisional Secretariat (DS) areas. Each DS area is divided to many Census Blocks, and each Census Block consists of many households. Primary sampling unit (PSU):

The primary sampling unit (PSU) was a Divisional Secretariat (DS) area. Out of 331 DS areas available, 80 DS divisions were selected using proportionate to the size (PPS) sampling. Secondary sampling unit (SSU): A census block was considered as a SSU. From each DS division (PSU), six secondary sampling units (SSU) were selected using the proportionate to the size (PPS) sampling technique. Therefore, a total of 480 SSUs or census blocks were selected from 80 PSUs. Tertiary sampling unit (TSU):  Number of houses in each census block depends on the area density and the population density in each DS division. Tertiary sampling unit (TSU) was the household and 15 households from each CB by random systematic sampling by the Department Census and Statistics.  Therefore, a sample of 7200 (80x6x15) households were selected. In some instances, there were more than one household living in one house. People who are cooking and eating together were considered as one household.  Whenever there were more than one household in a house, one household was selected randomly to be included in the study. Selection of participants: Only one participant from each household was included in the survey. All the eligible members in the selected family were listed in descending order according to the age. Once this was done, these data was fed to the personal digital assistants (PDAs). The PDAs then automatically selected the eligible participant using the Kish method.”

*Source: Non Communicable Disease Risk Factor Survey Sri Lanka 2015. Available at: https://www.who.int/ncds/surveillance/steps/sri_lanka/en/.*

# **Sudan STEPS 2016**

“A four-stage cluster sampling design was implemented. The four sampling stages were; 1) selection of states from the six regions 2) selection of clusters (a cluster was a Popular Administrative unit), 3) selection of households and 4) selection of eligible individuals. First Stage (State): Administratively Sudan is divided into 18 states which are grouped in six regions, (North, East, Khartoum, Central, Kordofan and Darfur region (Table 1). States were randomly selected from each region. No geographical areas or populations were excluded from the sampling frame. Thus 11 states were selected, probability proportional to the size, to represent the six regions. A list of the selected states is shown in Table 2.1. Second Stage (Cluster PAU): The Popular Administrative Units (PAU) is the smallest geographically border unit. These were defined as the ‘cluster’ in the region. Clusters were randomly sampled from all PAUs, from both urban and rural strata, according to probability proportional to size in each state, and urban/rural distribution. The PAUs inaccessible due to security conditions were not excluded from the sampling frame, because within certain areas the security status was continuously changing. However, it was planned that if a PAU was found to be inaccessible at survey time, it should be replaced by the nearest accessible unit. However, no replacement was required during this survey. Third Stage (Household): Within the selected PAUs, all households (HH) were included in the sampling frame. Accordingly (HH) were selected using systematic random methods. Fourth Stage (Individual): The members of the household were first listed in the mobile application (customized software). The inclusion criteria for the listed members were: all individuals aged between 18 to 69 years, from both sexes, irrespective of his health status and living in the selected household for a minimum of 6 weeks. The application was then run and it randomly selected the individual who will be selected to participate in the study.”

*Source: Sudan STEPwise Survey for Non-Communicable Diseases Risk Factors 2016 Report. Available at: https://www.who.int/ncds/surveillance/steps/sudan/en/.*

**Swaziland STEPS 2014**

“A multi-stage cluster sampling design was applied. The survey covered all the four regions of the country. The size of the country and the distances between the regions and communities made it possible for the survey to sample a population representing all the 4 regions. The multi-stage sampling procedure was implemented in the following procedural steps:

Stage 1: All four regions were included as a sampling frame of our Primary Sampling Unit (PSU).The number of the PSUs at this stage ensured precision in the survey estimates and as a result 216 PSUs were selected using probability proportional to size sampling.

Stage 2: The second stage of cluster sampling procedure entailed listing, sorting and random systematic sampling of the Secondary Sampling Units (Households) within the PSUs selected in stage1 where 20 households were selected from each PSU. Based on census data, only households with eligible participants were systematically sampled through random systematic sampling.

Stage 3: At this level, all the eligible participants within a household were sequentially listed into the PDAs and only one participant per household was randomly sampled using KISH method built into the PDAs. The KISH method is a widely used technique that uses a pre-assigned table of random numbers to identify the person to be interviewed.”

*Source: WHO STEPS: Noncommunicable Disease Risk Factor Surveillance Report Swaziland 2014. Available at: http://www.who.int/chp/steps/swaziland/en/.*

# **Tajikistan STEPS 2016**

“A multi-stage cluster sample of households. One individual within the age range of the survey was selected per household.”

*Source: NCD Microdata Repository. Study Description. Available at: https://extranet.who.int/ncdsmicrodata/index.php/catalog/270/study-description#page=sampling&tab=study-desc*

**Tanzania STEPS 2012**

“The STEPS survey in the United Republic of Tanzania was a population-based survey of adults aged 25-64. The study used both multistage cluster and random probability sampling procedures. Fifty of 119 total districts were randomly selected as primary sampling units (PSUs). Within these PSUs, enumeration areas (EAs) of > 50 households were randomly selected. Any EA with < 50 households was merged with a neighboring EA. Within the EAs, households were randomly selected from a list of all eligible households in the EA. A total of 5762 adults participated in the Tanzania STEPS survey. Within each selected household, the Kish method was used to select the STEPS participant. This procedure was followed until the predetermined sample was obtained for the enumeration area. The response rate for this survey was 94.7%.”

*Source: Tanzania STEPS Survey Report. Available at: http://www.who.int/chp/steps/UR_Tanzania_2012_STEPS_Report.pdf?ua=1*

**Timor Leste STEPS 2014**

“Note: Data from Census 2010 were used for all sampling considerations. Even though planning and mapping for 2015 Census is ongoing, data from the Census will only be available after July 2015.

STEP 1: Selection of Enumeration Area

(1) List of EA with number of HH by district for Census 2010 was obtained from the Directorate of Statistics. There are 1826 EAs in Timor-Leste. Out of these, 150 EAs were selected.

(2) The number of EAs to be selected from each district was based on their proportion in the country’s population as per Census 2010.

(3) The numbers of Households (HH) per EAs varied from 0 to more than 300. Therefore, probability proportion to size (PPS) was used.

(4) For each district, the EAs were arranged in ascending order of HH size.

(5) Sampling interval was obtained by dividing the total number of HH in the district by the number of EA to be selected from that district.

(6) A random number was generated between one and the sampling interval for that district, using tools available at random.org.

(7) The EA where that random number fell was the first EA to be selected.

(8) Subsequently, the sampling interval was added to the random number and the EA where this new number fell was selected. For the next number, the sampling interval was added to the number and so on, till the population of HH was exhausted or target number of EA achieved.

(9) This was done separately for each district.

(10) The final list was compiled and had 150 EAs. These are spread over about 125 sucos.

STEP 2. Selection of Households in an Enumeration Area

Listing the house numbers to be visited

(1) It was decided to use the 2010 HH size of each EA. Based on past experience, it was expected that the increase would be on an average about 4–5%.

(2) The list of households to be selected by enumerators was decided centrally.

(3) Sampling interval was calculated by dividing the total number of households in the EA by 18.

(4) The first HH number was selected randomly by reading the last two digits of a currency note. If the number represented by the two digits was more than 18, the last digit was taken into consideration. For each EA, a different currency note was used. This could also be done it by using the tool at random.org. or by draw of lots.

(5) The subsequent HH are identified by adding the sampling interval as was done for selection of EA.”

*Source: Timor-Leste STEPS Survey Report, [online] at* [*http://www*](http://www)*.who.int/entity/chp/steps/Timor-Leste_2014_STEPS_Report.pdf?ua=1*

**Togo STEPS 2010**

“Those included in this survey are male or female subjects, living in urban or rural areas, aged 15 to 64 on the day of the survey, residing in the enumeration area for at least 6 months and having given their informed consent to participate in this study. [...] Three hundred clusters were randomly selected in a systematic draw with probability proportional to the size of the cluster (number of households) in the 4620 areas of enumeration of the DGSCN (General Directorate of Statistics and National Accounts) sampling frame. In order to obtain the 4,800 households at the rate of 1 individual / household, 16 households per cluster were randomly selected at the second stage of survey. In each of the selected households, one individual was selected as a survey participant via the Kish Method. A household was defined as the group of persons, who regularly share the main meal (regardless of their relationship). Households were not replaced in the event of a refusal or two unsuccessful visits to the eligible person selected by Kish's method. If the selected person was unwell or not present at the time of the interview, the investigators either tried to find a new appointment or searched for the respondent.”

*Source, translated from: WHO: The Final Report on the Togo STEPS Survey 2010. Available at: http://www.who.int/chp/steps/2010STEPS_Report_Togo_FR.pdf?ua=1.*

# **Tonga STEPS 2017**

“The STEPS survey in Tonga was a population-based survey of adults aged 18-69 years. A Multi-stage clustering sample design was used to produce representative data for that age range. A total of 3858 adults participated in this Tonga STEPS survey. The overall response rate was 85.7%.”

*Source: Tonga STEPS Survey 2017 Fact Sheet. Available at: https://extranet.who.int/ncdsmicrodata/index.php/catalog/713*

# **Turkmenistan STEPS 2018**

„The two-stage probability sampling method with the use of stratification and selection procedures for each stage of sampling was used for forming the sampling frame for the STEPS study.

Geographical coverage - all regions of Turkmenistan: Akhal, Balkan, Dashoguz, Lebap and Mary velayats and the city of Ashgabat (the capital), which corresponds to the national administrative-territorial division. Stratification was carried out to ensure that the sampling population was evenly distributed throughout the country. Taking into account the division of each velayat into urban and rural population, a total of 11 strata were identified (Ashgabat city - only urban strata, in velayats - 10 strata). The total sample size was distributed in proportion to the number of households by strata.

Sample Formation.

The primary sampling units (PSUs) were physician sites that were selected from each stratum through systematic sampling procedures with probability proportional to size (PPS). The first stage of sampling was completed by selecting the required number of physician sites in each of the 6 regions separately for urban and rural strata (216 clusters in total).

The second stage involved selecting 20 households in each enumeration area using random systematic selection procedures. As a result, 4,320 households were selected (2,160 in urban areas and 2,160 in rural areas) (Table 1).

Household-level participants were selected using the eSTEPS mobile app to randomly select one eligible member (age 18-69) in each household.

The Kish method was used to randomly select one person aged 18-69 from each household.”

*Source: Department of Public Health, Ministry of Health (2020). Non-communicable disease Risk Factors: Turkmenistan STEPS Survey 2018*

# **Tuvalu STEPS 2015**

“The Tuvalu STEPS Survey was a population based cross-sectional survey of 18-69 year olds.”

*Source: NCD Microdata Repository. Study Description. Available at: https://extranet.who.int/ncdsmicrodata/index.php/catalog/639/study-description#page=sampling&tab=study-desc*

**Uganda STEPS 2014**

“Uganda has a total population of 34.9 million people, approximately 43% of which are adults aged 18 years or older [14]. The survey covered the whole country, and a three stage sampling design was used to select participants. The sampling procedure utilized the Uganda Bureau of Statistics (UBOS) master sampling frame of Enumeration Areas (EAs) that had just been demarcated throughout the country in preparation for the 2014 population and housing census. Each EA included 150–200 households. In the first stage, a random sample of 350 out of 78,950 EAs was selected with selection probability proportional to the size (PPS) of the number of households in the EAs. The EAs were stratified across the four regions of Uganda namely: Central, Eastern, Northern and Western region; and were selected with separate estimates for rural and urban areas. Urban areas were defined as EAs within government designated urban areas, or those within other geographic divisions with population density of more than 1000 per square kilometer.

After selecting the 350 EAs, trained teams of UBOS staff were dispatched throughout the country to list the households and their household heads within the 350 EAs. A household was defined as a group of individuals that usually shared meals together, and had a household head who usually made major decisions for the household. In the second stage of sampling, 14 households were randomly selected from the listed households in each of the sampled EAs.

Research Assistants (RA) that had received a five-day training on procedures and administration of the STEPs tool, enumerated eligible household members who were recorded in Personal Digital Assistants (PDA), which was then used to randomly select one subject for inclusion in the survey giving a total sample of 4900. Eligible subjects were household members aged 18 to 69 years, who had resided in the sampled households for at least six months preceding the date of interview.”

*Source: Guwatudde D, Mutungi G, Wesonga R, Kajjura R, Kasule H, Muwonge J, et al. (2015) The Epidemiology of Hypertension in Uganda: Findings from the National Non-Communicable Diseases Risk Factor Survey. PLoS ONE 10(9): e0138991. doi:10.1371/journal.pone.0138991.*

**Ukraine Demographic and Health Survey 2007**

“The sample was designed to allow detailed analysis of indicators—including the estimation of fertility, abortion and infant/child mortality rates at the national level and for urban and rural areas. Many indicators can also be estimated for the following five domains or geographical areas: North, Central, South, East, and West. Each domain consists of a few administrative divisions out of the total 27 administrative regions existing in Ukraine (24 regions, the capital city Kyiv, the city of Sevastopol, and the Autonomous Republic of Crimea), except for the clusters affected by the Chernobyl disaster and are uninhabitable.^1^

- North: the city of Kyiv, and the regions of Kyiv, Zhytomyr, Sumy and Chernihiv;
- Central: the regions of Cherkasy, Poltava, Kirovohrad and Vinnytsia;
- South: the Autonomous Republic of Crimea, the city of Sevastopol’ and the regions of Odesa, Mykolaiv and Kherson;
- East: the regions of Dnipropetrovs’k, Donets’k, Zaporizhzhia, Luhans’k, and Kharkiv;
- West: the regions of Ivano-Frankivs’k, Khmel’nyts’kyi, Chernivtsi, L’viv, Rivne, Ternopil’, Volyn’ and Zakarpattia.

A representative sample of households was selected for the 2007 UDHS. The sample was selected in two stages. In the first stage, 500 clusters were selected in Kyiv and the 26 other administrative divisions from the list of enumeration areas in the master sample frame of the 2001 Ukraine Population Census (SSC 2003a). In the second stage, a complete listing of households was carried out in each selected cluster. Households were then systematically selected from each cluster for participation in the survey. This design resulted in a final sample of 15,004 households selected. All women age 15-49 who were either permanent residents of the selected households or visitors present in the household the night before the survey were eligible to be interviewed. In addition, all men age 15-49 in one-half of the selected households were eligible to be interviewed if they were either permanent residents or visitors present in the household the night before the survey. Interviews were completed for 6,841 women and 3,178 men.”

Footnote 1 “One cluster was originally selected from the Chernobyl area and was replaced.”

*Source: Ukrainian Center for Social Reforms, State Statistical Committee, Ministry of Health, Macro International Inc. 2008. Ukraine Demographic and Health Survey 2007. Kyiv, Ukraine, Maryland, USA. Available at: https://dhsprogram.com/pubs/pdf/FR210/FR210.pdf*

# **Uruguay STEPS 2013-2014**

“The survey was conducted on a sample representative of the population 15 to 64 years old residing in localities urban areas of Uruguay, of 10,000 and more inhabitants. A multistage probability sampling was carried out, with stratification by conglomerates without replacement. As a sampling frame, we used the resulting address directory from the last national census (INE 2011). […] In stage 1, prior to the call for bids, it was proposed to have at least 150 cases in each of the 12 locations in the Interior and 1,800 for Montevideo (consisting of a single locality). In this way, there were a total of 3,600 cases in a sample without replacement. The localities of the Interior, prior to the draw, were regrouped into strata that respond to socioeconomic and geographical characteristics, which were determined by the INE and used in the sampling designs of the surveys carried out by this institute, particularly the Continuous Survey of Homes (ECH). The 12 original strata (five from Montevideo, one from the metropolitan and six from the rest of the Interior) were regrouped into 6 strata such as shown in table 3. In these new strata stipulated for the first stage, 12 locations distributed proportionally to the weight of each new stratum. Localities within each stratum were selected with probability proportional to the number of inhabitants that each registers.”

*Source: Translated from: Uruguay Ministry of Health. 2ª Encuesta Nacional de Factores de Riesgo de Enfermedades No Transmisibles.*

**Vanuatu STEPS 2011**

“The survey used a cluster sampling design where the primary sampling unit was enumeration area (EA) and the secondary sampling unit was households. All 6 provinces in Vanuatu were included in the survey. One hundred and thirteen (113) EAs were randomly selected proportion to the size of the EA from a total of 411 EAs. Forty four (44) households were then randomly selected in each EA proportional to the number of households in each EA. The selection of participants within each household was done using the Kish method. The total number of households selected by combined Enrolment Areas was 4,972.”

*Source: Vanuatu NCD Risk Factors STEPS Report. Available at https://www.fao.org/fileadmin/templates/agphome/documents/horticulture/WHO/fiji/steps/Vanuatu_STEPS_Report_-_Final_200513.pdf*

# **Venezuela EVASCAM 2014-17**

A multi-stage stratified sampling method was used to selecta representative sample of the general population of Venezuela. 4454 women and men, aged 20 years and older, were recruited from randomly selected samples in the eight regions of Venezuela. Initially, 23 cities (1st stage) from the eight regions – one to four cities per region – were chosen. Each selected city was stratified by municipalities. Two municipalities (2nd stage) in each city, then two parishes (3rd stage) in each municipality, and finally two locations (4th stage) in each parish, were randomly selected. In the 5thstage, mappings and censuses of each location delimited the streets or blocks (primary sampling units) and selected the households to visit. Actual household visits were conducted in the 6th stage. The visits to households started from number 1 onwards skipping every two houses. That is, the household visited were 1, 4, 7, 10, 13, 16and so on. If the number of people required after covering all households of this sequence was not achieved, the sampling continued on households 2, 5, 8, 11, and so on, until obtaining the number of subjects required to complete the sample from that sector.

*Source: information provided by the survey team.*

# **Vietnam STEPS 2015**

“The sampling of STEPS was done in as part of the sampling for the (GATS) conducted in combination manner to save time and resources for these two surveys.  Applied the multi-stages complex sampling process, the sampling process done by GSO was as follow:  Sampling of clusters (EA) In the first stage of sampling, the primary sampling unit (PSU) was an enumeration area (EA). There are about 170,000 EAs in the whole Viet Nam and the average number of households in each EA is different between urban and rural areas. An average number of households in an urban EA and a rural EA is 133 households and 120 households, respectively.  Sample of EAs were selected from the master sample frame. The master sample frame was a cluster frame made by the GSO based on the frame of Population and Housing Census 2009 and updated with data of 2014. Based on the Population and Housing Census data 2009, GSO prepared a 15% of master sample to serve as a national survey sampling frame. The master sample frame contains 25,500 enumeration areas (EAs) from 706/708 districts of Viet Nam (2 island districts were excluded from the GSO master sample frame). The master sample frame of GSO was divided by two stratification variables: urbanization (1 = urban; 2 = rural) and district group (1 = district/town/city of province; 2 = plain and coastal district; 3 = mountainous, island district). It means that the master sample frame was divided into 6 sample frames or 6 strata. The probability proportional to size (PPS) sampling method was used to select sample of EAs from 6 strata of master sample frame. The final sample of GATS included 315 EAs in the urban and 342 EAs for the rural. From these 657 EAs, 315 EAs were systematically selected for STEPS.  Sampling of households: At the second stage of sampling, 10% households in each EA were selected. Thus, 15 households from the selected urban EA and 14 households from the selected rural EA were chosen using simple systematic random sampling. The total households for STEPS 2015 were 4,651 households.  Sampling of individuals: One eligible person is then randomly selected from each selected household for the STEPS 1 interview. The selection of individual is automatically done by the PDA program after eligible household members are entered into the PDA.”

*Source: National Survey on the Risk Factors of Non-Communicable Diseases (STEPS) Viet Nam 2015. Available at: https://www.who.int/ncds/surveillance/steps/viet_nam/en/.*

# **Zambia STEPS 2017**

“To ensure that the sample reflected the entire country of Zambia, a multi-stage cluster sampling technique was used to select a nationally representative sample of adults in Zambia aged 18 to 69 years. It was decided to utilize the household listing from the Zambia Population- Based HIV Impact Assessment (ZAMPHIA) - a household-based national survey that was conducted between March and August 2016 in order to measure the status of Zambia’s national HIV response. ZAMPHIA offered the most pragmatic up to date and accessible national household listing to be used as the sampling frame for this survey. The ZAMPHIA survey included 60,581 households drawn from 1,103 clusters referred to in this report as standard enumeration area (SEA). Thus the sample drawn for the STEPS survey was a subsample of the households selected for the ZAMPHIA survey. In the first stage of sampling, SEAs were selected from each province using probability proportional to size (PPS). In the second stage, 15 households in rural SEAs and 20 households in urban SEAs were selected systematically using appropriate sampling interval based on the number of households in that SEA. These households constituted the final list of households for the STEPS survey prepared for the field investigators (FI). In the third stage, while the FI approached the household and sought consent, all eligible members in the household were entered into the Android-based devise used for the survey. The device then selected one member from the eligible members using a simple random sampling technique. The selected member was then interviewed having gone through the ethical process of consent after being provided with information on the survey. If the selected member was not available, a scheduled visit was made. If the selected member could not be reached after two scheduled visits he or she was considered as non-response. There was no replacement strategy so as to maintain the integrity and representativeness of the sample.”

*Source: Zambia STEPS for For Non Communicable Diseases Risk Factors (2017). Available at: https://www.who.int/ncds/surveillance/steps/zambia/en/.*

The PNS will integrate the SIPD, which will make it possible to relate the information collected with other researches, such as the PNAD and the Household Budget Survey (POF) at different levels of geographic aggregation.”

*Source, translated from: Pesquisa Nacional de Saúde. Plano de Amostragem. 2010. Available at: https://www.pns.icict.fiocruz.br/index.php?pag=planoamostragem [accessed May 11 2018]*

**Zanzibar STEPS 2011**

“The survey took place in June and July 2011, followed by data cleaning and analysis. One Principal Investigator and five assistant researchers coordinated the survey on site, checked completed questionnaires daily, and organised logistics. The six data collection teams consisted each of six interviewers, one supervisor, one laboratory technician and one driver. Interviewers were either health care workers or professional interviewers familiar with household surveys such as DHS. The sample size was calculated to be 2800 participants. Each interviewer did on average 3 – 4 interviews a day and was assisted on site by local village guides.

Study design:

Cross-sectional population based survey with a sample of a sufficient size with a power to determine the proportion of adults that are exposed to selected risk factors associated with NCDs; including those having raised BP, FBG or blood lipids, had experienced injuries or traumas in recent times, and/or were mentally unwell (anxiety, depression), as well as linking these conditions with one another and with the sociodemographic and economic information obtained.

Study population:

People reported to be permanent residents (spending on average maximum 3 nights per week outside the house, and not holding an address in another place) in the selected households and fulfilled the inclusion criteria were enrolled into the survey. A person could only appear once in the study. Therefore we classified a husband practicing polygamy to be listed in the household of his first wife but not to be a member in the household of the following wives.

Sampling Frame:

The target population is the entire population in Zanzibar whereby the whole of Zanzibar was selected as the survey site, and hence all districts included. The total population is estimated to be 1.2 million distributed unevenly between 10 districts. The sampling frame represented the entire population in Zanzibar.

Sampling Technique:

The sampling strategy used is a multi-stage cluster sampling with stratification. The ten districts are considered as different strata, and the total number of primary sampling units, PSU, is allocated proportionately across all strata. Each district is divided into smaller clusters. These clusters are the geographical and administrative units called Shehia11. The Shehia are divided into smaller clusters called zones (also called mitaa, vitongoji, or vijiji) which typically consist of 100-300 households. Zones smaller than that were merged to make up one larger cluster, and zones much larger were split in smaller clusters.

At the first stage clusters were selected using Simple Random Selection, SRS, from the list of clusters (Shehia) within each district. At the second stage clusters (zones) were randomly selected using probability proportionate to size (PPS). At the third stage households were randomly selected from the household lists provided by the administrative leader of the Shehia.

The two last stages of sampling was done using the software STEPSsampling.xls from WHO. Finally participants were selected from the household using Kish method. The household lists were complete and included households with no eligible participants for the survey. Therefore an extra 7 households were sampled at third stage in each cluster for replacement in case a selected household had no eligible participants and had to be changed. This was done before data collectors went to the cluster. “

Resources allowed for 100 PSU which was why 2800/100 = 28 households were selected from each PSU (and disproportionate from each SSU).

*Source: Zanzibar NCD Survey Report. Available at: https://extranet.who.int/ncdsmicrodata/index.php/catalog/621/related-materials*

***Supplemental References***

1. Global Health & Population Project on Access to Care for Cardiometabolic Diseases (HPACC). Expanding access to newer medicines for people with type 2 diabetes in low-income and middle-income countries: a cost-effectiveness and price target analysis. Lancet Diabetes Endocrinol 2021;9:825–836
2. Rodbard HW, Lingvay I, Reed J, et al. Semaglutide Added to Basal Insulin in Type 2 Diabetes (SUSTAIN 5): A Randomized, Controlled Trial. J Clin Endocrinol Metab 2018;103:2291–2301
3. Rosenstock J, Jelaska A, Zeller C, et al. Impact of empagliflozin added on to basal insulin in type 2 diabetes inadequately controlled on basal insulin: a 78-week randomized, double-blind, placebo-controlled trial. Diabetes Obes Metab 2015;17:936–948
4. Edridge CL, Dunkley AJ, Bodicoat DH, et al. Prevalence and Incidence of Hypoglycaemia in 532,542 People with Type 2 Diabetes on Oral Therapies and Insulin: A Systematic Review and Meta-Analysis of Population Based Studies. PLoS One 2015;10:e0126427
5. Xie X, Guo J, Bremner KE, Wang M, Shah BR, Volodin A. Review and estimation of disutility for joint health states of severe and nonsevere hypoglycemic events in diabetes. J Comp Eff Res 2021;10:961–974
6. Aroda VR, Bain SC, Cariou B, et al. Efficacy and safety of once-weekly semaglutide versus once-daily insulin glargine as add-on to metformin (with or without sulfonylureas) in insulin-naive patients with type 2 diabetes (SUSTAIN 4): a randomised, open-label, parallel-group, multicentre, multinational, phase 3a trial. Lancet Diabetes Endocrinol 2017;5:355–366
7. Zinman B, Wanner C, Lachin JM, et al. Empagliflozin, Cardiovascular Outcomes, and Mortality in Type 2 Diabetes. N Engl J Med 2015;373:2117–2128
8. Bettge K, Kahle M, Abd El Aziz MS, Meier JJ, Nauck MA. Occurrence of nausea, vomiting and diarrhoea reported as adverse events in clinical trials studying glucagon-like peptide-1 receptor agonists: A systematic analysis of published clinical trials. Diabetes Obes Metab 2017;19:336–347
9. Vetter ML, Johnsson K, Hardy E, Wang H, Iqbal N. Pancreatitis Incidence in the Exenatide BID, Exenatide QW, and Exenatide QW Suspension Development Programs: Pooled Analysis of 35 Clinical Trials. Diabetes Ther 2019;10:1249–1270
10. Meier JJ, Nauck MA. Risk of pancreatitis in patients treated with incretin-based therapies. Diabetologia 2014;57:1320–1324
11. Center for Evaluation of Value and Risk in Health (CEVR). Global Health Cost Effectiveness Analysis Registry [Internet], 2019. Available from:<http://ghcearegistry.org/orchard/cite-the-gh-cea-registry>
12. Leiter LA, Mallory JM, Wilson TH, Reinhardt RR. Gastrointestinal safety across the albiglutide development programme. Diabetes Obes Metab 2016;18:930–935
13. Castoldi L, De Rai P, Zerbi A, et al. Long term outcome of acute pancreatitis in Italy: results of a multicentre study. Dig Liver Dis 2013;45:827–832
14. Dave CV, Schneeweiss S, Patorno E. Comparative risk of genital infections associated with sodium-glucose co-transporter-2 inhibitors. Diabetes Obes Metab 2019;21:434–438
15. Colacci M, Fralick J, Odutayo A, Fralick M. Sodium-Glucose Cotransporter-2 Inhibitors and Risk of Diabetic Ketoacidosis Among Adults With Type 2 Diabetes: A Systematic Review and Meta-Analysis. Can J Diabetes 2022;46:10-15.e2
16. Dawwas GK, Flory JH, Hennessy S, Leonard CE, Lewis JD. Comparative Safety of Sodium-Glucose Cotransporter 2 Inhibitors Versus Dipeptidyl Peptidase 4 Inhibitors and Sulfonylureas on the Risk of Diabetic Ketoacidosis. Diabetes Care 2022;45:919–927
17. Hooton TM, Bradley SF, Cardenas DD, et al. Diagnosis, prevention, and treatment of catheter-associated urinary tract infection in adults: 2009 International Clinical Practice Guidelines from the Infectious Diseases Society of America. Clin Infect Dis 2010;50:625–663
18. Desai D, Mehta D, Mathias P, Menon G, Schubart UK. Health Care Utilization and Burden of Diabetic Ketoacidosis in the U.S. Over the Past Decade: A Nationwide Analysis. Diabetes Care 2018;41:1631–1638
19. Cheong AJY, Teo YN, Teo YH, et al. SGLT inhibitors on weight and body mass: A meta-analysis of 116 randomized-controlled trials. Obesity (Silver Spring) 2022;30:117–128
20. Castellana M, Cignarelli A, Brescia F, et al. Efficacy and safety of GLP-1 receptor agonists as add-on to SGLT2 inhibitors in type 2 diabetes mellitus: A meta-analysis. Sci Rep 2019;9:19351
21. Jia H, Zack MM, Thompson WW. Population-Based Estimates of Decreases in Quality-Adjusted Life Expectancy Associated with Unhealthy Body Mass Index. Public Health Rep 2016;131:177–184
22. Perkovic V, Tuttle KR, Rossing P, et al. Effects of Semaglutide on Chronic Kidney Disease in Patients with Type 2 Diabetes. N Engl J Med 2024;0:null
23. Rådholm K, Wu JH, Wong MG, et al. Effects of sodium-glucose cotransporter-2 inhibitors on cardiovascular disease, death and safety outcomes in type 2 diabetes - A systematic review. Diabetes Res Clin Pract 2018;140:118–128
24. Nuffield Department of Population Health Renal Studies Group, SGLT2 inhibitor Meta-Analysis Cardio-Renal Trialists' Consortium. Impact of diabetes on the effects of sodium glucose co-transporter-2 inhibitors on kidney outcomes: collaborative meta-analysis of large placebo-controlled trials. Lancet 2022;400:1788–1801
25. Iwase M, Ide H, Ohkuma T, et al. Incidence of end-stage renal disease and risk factors for progression of renal dysfunction in Japanese patients with type 2 diabetes: the Fukuoka Diabetes Registry. Clin Exp Nephrol 2022;26:122–131
26. Finne P, Groop P-H, Arffman M, et al. Cumulative Risk of End-Stage Renal Disease Among Patients With Type 2 Diabetes: A Nationwide Inception Cohort Study. Diabetes Care 2019;42:539–544
